# Progress in family planning in Sierra Leone: a mixed-methods case study

**DOI:** 10.1136/bmjgh-2024-018775

**Published:** 2026-06-09

**Authors:** Philip S Amara, Mohamed Kanu, Alhassan Kanu, Francess Fornah, Clifford Kelfa Kamara, Joan H Shepherd, Tom Sessay, Francis L Moses, Sartie Kenneh, Hina Najmi, Abeer Mian, Mishaal Zulfiqar, Sacha St-Onge Ahmad, Jen Kidwell Drake, Zahid Ali Memon, Zulfiqar Ahmed Bhutta

**Affiliations:** 1 University of Michigan, Ann Arbor, Michigan, USA; 2 Center for Community and Public Health Improvement, Freetown, Sierra Leone; 3 Centre for Community & Public Health Improvement (CPHI), Freetown, Sierra Leone; 4 Faculty of Nursing and Midwifery, College of Medicine and Allied Health Sciences, University of Sierra Leone, Freetown, Sierra Leone; 5 Directorate of Policy, Planning and Information, Government of Sierra Leone, Ministry of Health and Sanitation, Freetown, Sierra Leone; 6 Directorate of Reproductive and Child Health, Government of Sierra Leone, Ministry of Health and Sanitation, Freetown, Sierra Leone; 7 Ministry of Health and Sanitation, Government of Sierra Leone, Freetown, Sierra Leone; 8 The Aga Khan University, Karachi, Sindh, Pakistan; 9 Department of Community Health Sciences, The Aga Khan University, Karachi, Pakistan; 10 Aga Khan University, Karachi, Pakistan; 11 The Hospital for Sick Children, Centre for Global Child Health, Toronto, Ontario, Canada; 12 Gates Venture, Seattle, Washington, USA; 13 Department of Community Health Sciences, Aga Khan University, Karachi, Pakistan; 14 Center of Excellence in Women and Child Health, Aga Khan University, Karachi, Pakistan

**Keywords:** Global Health, Health policies and all other topics, Health policy, Health systems, Public Health

## Abstract

**Introduction:**

Expanding access to voluntary family planning is a global health priority, yet progress has been uneven across low-income and middle-income countries. Despite being a fragile state with historically high fertility and limited health resources, Sierra Leone has achieved one of the fastest increases in modern contraceptive prevalence in sub-Saharan Africa. This study explores the drivers of this progress through a mixed-methods case study.

**Methods:**

We analysed national-level data from urban and rural settings, and engaged diverse stakeholders including family planning professionals, healthcare providers, community leaders and service users. Quantitative data were drawn from Demographic and Health Surveys (DHS 2008, 2013 and 2019), and United Nations (UN) Population Division estimates (1990–2022). Qualitative insights were obtained through 33 key informant interviews and 12 focus group discussions. A systematic review of peer-reviewed articles, policies and programme documents was conducted to assess the broader implementation context.

**Results:**

Between 2008 and 2019, modern contraceptive prevalence among women aged 15–49 rose from 8.2% to 23.9% (+15.7 percentage points). Among married women, gains were larger in those aged 15–24 years (+12.6) than in women aged 25–49 years (+10.0). Unmarried women maintained substantially higher use, with gains of +19.5 and +16.9 points, respectively. Key drivers included expanded knowledge, higher education, delayed marriage and outreach by fieldworkers. Political commitment—particularly integration into the Free Health Care Initiative (FHCI)—together with women’s empowerment policies, donor and non-governmental organisation (NGO) support, media and community engagement improved access, awareness and autonomy.

**Conclusions:**

Sierra Leone’s progress demonstrates the importance of holistic approaches combining political commitment, external financing, cross-sector partnerships and community engagement. Government leadership was most evident in integrating family planning into the Free Health Care Initiative, while educational and gender-sensitive strategies proved particularly effective. These drivers consolidated gains and offer lessons for fragile, high-fertility settings.

**WHAT IS ALREADY KNOWN ON THIS TOPIC**

- Sierra Leone has experienced one of the fastest increases in modern contraceptive prevalence in sub-Saharan Africa since 2008, with national surveys documenting steady gains.
- Despite this progress, inequalities remain by age, residence, education and region, with adolescents and young women continuing to face disproportionately high unmet need.
- Exposure to family planning messages through mass media supports contraceptive uptake, while persistent behavioural and social barriers—including low literacy, limited negotiation capacity and stigma—still constrain access for some groups.

**WHAT THIS STUDY ADDS**

- This study provides the first comprehensive evaluation of Sierra Leone’s family planning progress, documenting how modern contraceptive prevalence rate rose from 4.5% in 2001 to 29% in 2023. It highlights the role of sustained political commitment alongside post-war gender and health policies, youth-focused demand generation and education initiatives in driving this progress. By linking these policy commitments with strengthened service delivery, supply chain improvements and robust external partnerships, the study offers new insights into why Sierra Leone stands out as a reference case for family planning uptake in sub-Saharan Africa.

**HOW THIS STUDY MIGHT AFFECT RESEARCH, PRACTICE OR POLICY**

- The findings could influence policy by reinforcing the importance of galvanising commitments and bolstering community engagement and gender-focused strategies in family planning programmes.
- For practitioners, this study underscores the effectiveness of integrating educational outreach with community engagement to change norms and improve sexual and reproductive health outcomes.
- For researchers, the study establishes a model for using a comprehensive conceptual framework to examine the multifaceted effects of policies, programmes and financing on contraceptive use.

## Introduction

 Worldwide, maternal mortality remains a pressing challenge, and accelerating efforts to reduce the maternal mortality ratio (MMR)—particularly in sub-Saharan Africa—is imperative. Although the global MMR declined from 342 per 100 000 live births in 2000 to 223 in 2020, sub-Saharan Africa accounted for about 70% of maternal deaths that year, as shown in peer-reviewed syntheses^1^ and international agency reports.^2^ In Sierra Leone, a decade-long civil conflict (1991–2002) weakened the health system, resulting in some of the world’s highest maternal and child mortality rates.^3^ The crisis galvanised political will to prioritise reproductive and maternal health, with the Free Health Care Initiative (FHCI)—primarily aimed at reducing maternal and child mortality^4^—serving as the principal vehicle for nationwide delivery of family planning services, as documented in government reports^5^_’_.^6^ Recognised globally as a cost-effective intervention to reduce maternal mortality, family planning promotes healthy birth spacing and prevents unintended pregnancies, particularly among adolescents.^7^ Despite health shocks, socioeconomic challenges and persistent cultural barriers, Sierra Leone achieved a modern contraceptive prevalence rate (mCPR) of 29% among married women in 2023, according to Track20, a non-peer-reviewed analytic report.^8^ This marks noteworthy progress from the immediate postwar mCPR of 4.5% in 2001, positioning Sierra Leone as a stage 2 country on the contraceptive use growth curve.^8^ A multicountry study by The Exemplars in Global Health Family Planning Research Consortium recognised Sierra Leone as a positive outlier in family planning uptake.^9^ Recent national analyses similarly demonstrate that modern contraceptive use in Sierra Leone has increased since 2008,^10^ although significant inequalities persist.^11^

Despite progress in modern contraceptive uptake, there is limited peer-reviewed evidence systematically examining the underlying drivers of family planning trends in Sierra Leone. Most available information stems from grey literature, with published research largely confined to specific determinants, such as sociodemographic determinants,^10 11^ fertility-related disparities^12^ challenges and barriers, or service utilisation patterns.^13^,^15^ These studies, while informative, have not addressed how ecological, sociocultural, policy and programmatic factors—individually and synergistically—have influenced contraceptive uptake, nor have they adequately accounted for the complexities of Sierra Leone’s fragile, post-conflict environment. A recent multicountry analysis demonstrated that the strength of family planning programmes and higher levels of women’s education are strongly associated with increased contraceptive prevalence.^16^ However, comparable country-specific evidence from Sierra Leone remains limited.

Although comparative experiences from Ethiopia’s Health Extension Programmes and Rwanda’s Human Resources for Health (HRH) initiative demonstrate how community-based approaches and large-scale partnerships can strengthen health systems and accelerate progress in reproductive health, including family planning,^17 18^ little is known about how such strategies are adapted in fragile, post-conflict contexts such as Sierra Leone, which has faced multiple health shocks.

Against this backdrop, Sierra Leone offers a unique opportunity to investigate the factors shaping contraceptive uptake within a setting marked by heavy reliance on external financing, local policy reforms and extensive community engagement.

To address these gaps, this study systematically examines the ecological, sociocultural, individual, policy and programme determinants of contraceptive uptake in Sierra Leone, focusing on how demand-side and supply-side innovations and post-conflict strategies influence uptake. The findings aim to inform strategies for sustaining progress in Sierra Leone and to provide insights applicable to other countries facing similar challenges. This case study forms part of the Exemplars in Family Planning multicountry initiative, which uses a standard conceptual framework and methods to investigate drivers of progress.^19^

## Methods

### Study design

We employed a mixed-methods approach to examine drivers of mCPR in Sierra Leone, including a systematic scoping review, secondary quantitative analysis, programme and policy analysis, and qualitative research. The study adhered to the conceptual framework and methods described in the multicountry Exemplars in Family Planning initiative^19^ and followed Strengthening the Reporting of Observational Studies in Epidemiology (STROBE) cross-sectional reporting guidelines in line with best practices for observational research.^20^ Further details are provided in the published protocol.^19^

### Study setting

Sierra Leone, a West African country divided into five major provinces and 16 districts, had a population of 7 548 702 in 2021, with 50.6% females and an average household size of 4.25 persons. Population growth slowed to 1.04% per year from 2015 to 2021, with regional variation in household size and urban concentration, notably larger rural households in the East and denser populations in the urban Western region including Freetown.^21^

The population of interest for family planning consists of women aged 15–49 who have engaged in sexual intercourse in the previous 12 months, are not pregnant or menopausal, and do not self-report as infecund, regardless of parity. The total fertility rate (TFR) declined from 5.0 births per woman in 2008 to 4.3 in 2019 but remains nearly twice the replacement level.^12^ Fertility patterns display persistent disparities; women from the poorest households, those with no formal education and those residing in rural areas experience consistently higher fertility. For example, in 2019, TFR ranged from 5.1 in the northwestern province to 2.9 in the Western area.^12^ The median age at first birth among women aged 25–49 was 19.5 years^12^. Early childbearing remains common, with approximately 17% of women having their first sexual experience before age 15, and a substantial proportion of adolescents have unmet needs for modern contraception. Modern contraceptive use remains limited nationwide, although uptake is generally higher among women who are better educated, wealthier or urban-residing.^12^

Sierra Leone’s 1300 health facilities are predominantly state-run (94%). The health system operates under the Basic Package of Essential Health Services within four levels: maternal and child health posts (49%), community health posts (25.5%), community health centres (17.4%) and hospitals (4.4%).^22^ The skilled health worker density in 2017 was 6.4 per 10 000 population, well below the recommended minimum.^23^

### Systematic/scoping review

To explore drivers and contextual factors, we conducted a country-specific systematic scoping review (1999–2023) of determinants of family planning outcomes, focusing on mCPR, demand satisfied and unmet need. We systematically searched six databases—PubMed, Excerpta Medica Database (Embase), Psychological Information Database (PsycINFO), Cumulative Index to Nursing and Allied Health Literature (CINAHL), Web of Science and JSTOR—and identified grey literature from government and development partner sources. In total, 169 database records and 212 grey literature documents were screened using Covidence.^24^ Eligible studies included primary, representative data on relevant outcomes for reproductive-age populations. Experimental, qualitative and non-human studies were excluded. 12 peer-reviewed articles and 21 grey literature reports were included. Full search details are provided in online supplemental file 1, and study characteristics are summarised in onlinesupplemental files 2  3. A flow chart of study selection was provided in online supplemental figure 1.

### Quantitative analysis

#### Trend analysis

For the trend analysis, we compiled data from United Nations (UN) estimates (2000–2022) and Sierra Leone Demographic and Health Surveys (DHS).^25^,^28^ These data sources were used to describe national and subnational trends in mCPR, with results presented as simple proportions over time. Trends were mapped to major policy changes and programme milestones.

#### Decomposition analysis

The Oaxaca–Blinder decomposition analysis was used to identify factors contributing to changes in mCPR between 2008 and 2019, with mCPR as the dependent variable and independent variables including sociodemographic characteristics, family planning interventions, women’s agency and ecological factors. Analysis included women aged 15–49 who had sexual intercourse in the previous 12 months, were not pregnant or menopausal and did not self-report as infecund, regardless of parity. Further DHS definitions are available in the Guide to DHS Statistics (The DHS Programme, ICF Macro (Inner City Fund Macro International Inc, now ICF International).^29^ Hierarchical modelling explored determinants of mCPR growth, stratified by age group (15–49, 15–24, 25–49 years) and marital status. Variables with p values ≤0.25 were retained, with adjustments for confounders, stratified analyses and collinearity checks (variance inflation factor >10). Respondents with missing data were excluded to preserve representativeness and statistical power. The complete methods are described in online supplemental file 4.

### Programme and policy review

The desk review covered policy, programme and finance documents to map implementation processes, funding flows and key milestones. Sources included direct stakeholder requests and searches of online repositories. Data were extracted using a standard template to construct a timeline of key policies and programmes (1990–2022).

### Qualitative research

#### Participants and sampling

Qualitative data were collected through key informant interviews (KIIs) and focus group discussions (FGDs) with two stakeholder groups. Group one comprised national and district family planning professionals, public health officials, civil society organisations and development partners, purposively selected for their expertise. Group two included community stakeholders—adolescents and adults—sampled based on residence in areas with family planning services or user/provider experience. Districts were purposively selected for geographic diversity, mCPR variation and differences in programme implementation, ensuring high and low uptake districts and regionally balanced representation.

#### Data collection

Semistructured KIIs and FGDs were conducted between June and July 2024 in private settings. Tools were pretested and refined. Oral and written informed consent were obtained; confidentiality and voluntary participation were emphasised. Trained assistants, supervised by the fifth author, collected data, following a 2-day training on ethics and quality assurance. All interviews were audio-recorded, transcribed verbatim and anonymised. Interview guides are in online supplemental file 5.

#### Data analysis

Thematic analysis was conducted using MAXQDA (version 24), guided by the study’s conceptual framework. The coding framework was refined iteratively. Transcripts were coded into predefined and emergent categories; disagreements were resolved by discussion, and themes were developed to inform the analysis.

### Evidence synthesis and study organisation

This study formed part of the Exemplars in Family Planning multicountry initiative, following an established conceptual framework. Country selection was informed by mCPR and demand satisfied relative to the Human Development Index, classifying Sierra Leone as a positive outlier.^9^

Research was organised into four streams: (1) systematic scoping review, (2) quantitative analyses, (3) policy and programme review, and (4) qualitative research. The Sierra Leone and Exemplars teams jointly led the scoping and policy review, with quantitative analyses conducted by Aga Khan University and SickKids. Qualitative research was implemented locally and cross-validated by Exemplar partners.

Evidence synthesis used a narrative, multistage approach. Preliminary findings from each stream were discussed by both study teams and presented at an international conference, guiding instrument refinement and theme validation. Standardised formats (such as an Excel-based thematic matrix) supported cross-stream comparison. Synthesis drafts were iteratively reviewed in joint team sessions and monthly multicountry calls, with discrepancies resolved by consensus. Preliminary results were validated at a national workshop attended by over 100 stakeholders. Feedback was incorporated to enhance policy relevance and credibility.

Triangulation of findings is summarised in table 1, demonstrating consistency across research streams.

**Table 1:** Convergence of evidence across analytic streams for key factors influencing family planning outcomes in Sierra Leone

Themes	Source of evidence			
	Literature review	Decomposition analysis results	Key informant interviews and/or focus group discussions	Programme*/policy /finance review
Political commitment and advocacy	X	–	X	X
External financing and partnerships	X	–	X	X
Supply chain and service delivery	X	–	X	X
Community engagement on gender equality and women’s reproductive rights	X	–	X	X
Demand generation through education and awareness	X	X	X	X

* ‘Programme’ uses UK spelling.

–, theme not identified in this source; X, theme supported by evidence from this source.

## Results

### Systematic/scoping review

We conducted a scoping review of the literature on family planning and reproductive health in Sierra Leone, following the Preferred Reporting Items for Systematic Reviews and Meta-Analyses extension for Scoping Reviews (PRISMA-ScR) guidelines. The search covered studies published in English from 1990 to 2022 in PubMed, JSTOR, Embase (Excerpta Medica Database), MEDLINE (Medical Literature Analysis and Retrieval System Online), and Web of Science and included grey literature from government ministries, United Nations Population Fund (UNFPA), local non-governmental organisations (NGOs) and other development partners.

After screening 169 peer-reviewed and 212 grey literature records, 12 peer-reviewed articles and 21 grey literature documents were included. These comprised cross-sectional surveys, DHS, evaluations, programme reports and policy analyses.

Findings from these sources indicated that between 2008 and 2019, the mCPR among married women rose from 7% to 21%.^26 28^ Uptake was highest for injectables, implants and pills. Major determinants of mCPR included age, education, parity, marital status, urban residence, region, mass media exposure (especially to radio and mobile phone), health facility access, while at the broader sub-Saharan level, evidence highlights the importance of strong policy environments in driving contraceptive uptake.^13 15 16^ National strategies—such as costed implementation plans, the Basic Package of Essential Health Services and adolescent health guidelines—were consistently associated with increases in uptake. Partner support and multisectoral collaboration also played pivotal roles.

Persistent barriers included lower contraceptive use and access in rural and remote areas, contributing to ongoing urban–rural and regional disparities.^13 15^ Despite high national levels of method awareness, unmet need (around 25%), misconceptions about fertility, high discontinuation rates (notably among adolescents) and gaps in service quality remain significant challenges.^14^ Overall fertility declined, but ongoing gaps in knowledge and counselling were widely reported.^28^

### Quantitative analysis

#### Modern contraceptive prevalence and demand satisfied

Between 2008 and 2019, Sierra Leone’s mCPR increased, from 8.2% to 23.9% among all women and from 6.7% to 20.9% among married or in-union women (DHS table 2). The sharpest rise occurred after 2008. Gains were observed across both married and unmarried women, with mCPR among sexually active unmarried women aged 15–24 reaching 57.5% by 2013 and remaining high in 2019 (58.1%). Table 2 presents mCPR by marital status and age, with subgroup N calculated from original DHS reports or as weighted averages of DHS age and marital groups. Any discrepancies from totals reflect DHS denominator conventions, as described in the survey documentation. Demand satisfied by modern methods also reached 53.1% in 2019 for all women, and 45.4% for married women in 2019. Despite this progress, gains have slowed recently, with persistent challenges for rural, adolescent and less affluent women.^28^

**Table 2:** Modern contraceptive prevalence rate (mCPR, %) by age group and marital status, Sierra Leone, Demographic Health Survey (DHS) 2008–2019

Population group	2008 mCPR^*^	N 2008	2013 mCPR*	N 2013	2019 mCPR*	N 2019
All women (15–49 years)	8.2	7374	20.9	16 658	23.9	15 574
All women aged 15–24	7.9	2384	23.7	6561	24.9	6056
All women aged 25–49	8.3	4989	19.1	10 096	23.4	9519
Married/in-union (15–49 years)	6.7	5525	15.6	10 903	20.9	9715
Married/in-union, age 15–24	3.7	1171	11.8	2299	17.2	1842
Married/in-union, age 25–49	7.6	4353	16.6	8603	21.8	7873
Unmarried/sexually active, age 15–49†	24.5	551	56.3	2058	52.6	1987
Unmarried/sexually active, age 15–24†	26.6	343	57.5	1472	58.1	1309
Unmarried/sexually active, age 25–49†	21.1	208	53.3	586	42.1	678

Sample sizes by subgroup do not sum to overall totals due to DHS eligibility criteria, non-response, or analytic restrictions. For example, ‘all women aged 15–24’ and ‘25–49’ may not sum exactly to ‘all women 15–49’ if missing data or ineligible respondents are excluded in subgroup analyses; see DHS documentation for precise denominator definitions in each year. CIs are not presented because they are not included in the published DHS tables for these subgroups, and correct calculation requires access to the full survey dataset and design variables.

All prevalence rates and denominators are from DHS 2008 (Table 5.4), DHS 2013 (Table 7.2) and DHS 2019 (Table 7.3), or computed as described in Methods.

* mCPR=modern contraceptive prevalence rate (percentage of women currently using any modern contraceptive method), as reported in respective DHS surveys.

† Unmarried women defined as sexually active, not in union (self-reported sex in last 30 days. N=number of women in analytic (sub-)population per DHS table for each year and subgroup. All estimates use DHS sample weights and standard analytic methods. Calculated values for age subgroups represent weighted averages of individual 5-year cohorts; see Methods for details. For ‘unmarried’ categories, sample is limited to sexually active women (self-reported sex in last 30 days).

### Socio-economic, geographic and demographic inequities

Marked disparities persist. In 2019, mCPR was 25.8% among the wealthiest married women vs 15.8% for the poorest, and 25.8% among urban women compared with 18.1% in rural areas.^28^ Contraceptive use increased with education (28.3% for those with higher education, vs 16.7% with none) and parity (21.9% among women with five or more children, just 8.4% for those without children). Adolescents (15–19 years) had the lowest mCPR at 8.9% in 2019, reinforcing the unmet needs of this group.

### Method mix and fertility preferences

Injectables were most common (8.9% in 2019), with a rise in use of implants (6.8%), while pill use declined over time. Long-acting and permanent methods remained limited. The ideal number of children desired dropped from 5.0 in 2008 to 4.7 in 2019,^28^ echoing shifting fertility preferences seen among both women and men.

### Reproductive agency and family planning communication

Women’s autonomy in contraceptive decision-making steadily improved, with 49.7% of married women deciding alone in 2019, up from 32.1% in 2008.^26 28^ Early sexual debut remained high: 25.8% by age 15, 91.6% by 20 (2019). Exposure to family planning messages increased between 2008 (46.2%) and 2013 (59.3%) but declined sharply to 29.6% in 2019. By 2019, 53.3% of married women reported no exposure to family planning messages via radio, TV or print media.**26,28**

### Contact with family planning providers

Gaps in engagement persisted, with 83.0% of non-users of family planning having no discussion with providers in 2008, dropping to 57.7% in 2019.^26 28^ Among women who visited a health facility,the proportion who were spoken to about family planning rose from 13.5% to 36.6% over the same period. Contact by fieldworkers increased from 6.7% in 2008 to 23.8% in 2013 but then declined to 18.2% in 2019.^26^,^28^

### Informed choice and quality of counselling

Informed choice indicators improved markedly in 2013 but declined slightly by 2019. The share of users informed about side effects rose from 59.7% to 79.1% (2013), then fell to 77.1% (2019). The Method Information Index peaked at 73.6% in 2013 and settled at 70.9% in 2019.^27 28^

### Access and sources of contraceptive supply

Government-provided contraception use increased from 14.3% in 2008 to 30.1% in 2019, while private sector reliance dropped (11.7% to 5.8%) and other sources declined, reflecting greater public sector availability.^26 28^

### Barriers to contraceptive use

Among married women who reported no intention to use contraception in the future in 2008 (indicator not reported in later DHS), top reasons for non-use included spouse opposition (14.4%), personal opposition (13.5%), lack of method knowledge (11.3%), desire for more children or spacing (10.8%), fear of side effects (10.8%), religious concerns (9.3%) and health worries (3.4%). Cost, inconvenience and lack of access were rarely cited.^26^

### Unmet need for family planning

Unmet need declined from 28.0% in 2008 to 24.8% in 2019, with both spacing and limiting contributing to the trend.^26 28^ Urban and rural unmet need converged by 2019, but absolute levels remained high. Educational differences in unmet need persisted: women without education had lower unmet need (23.6%), while those with secondary schooling had higher needs for spacing, reflecting differences in fertility preferences across groups.

### Decomposition analysis of mCPR change

Oaxaca–Blinder decomposition (DHS 2008/2019) found mCPR gains driven by rising education, household wealth, urban residence and family planning information exposure. Changing fertility desires and access/method mix also played key roles. Urban and more educated women gained most, but equity gaps narrowed overall. Full model outputs are available in online supplemental file 2.

### Programme and policy review

#### Overview and timeline

**Figure 1:**
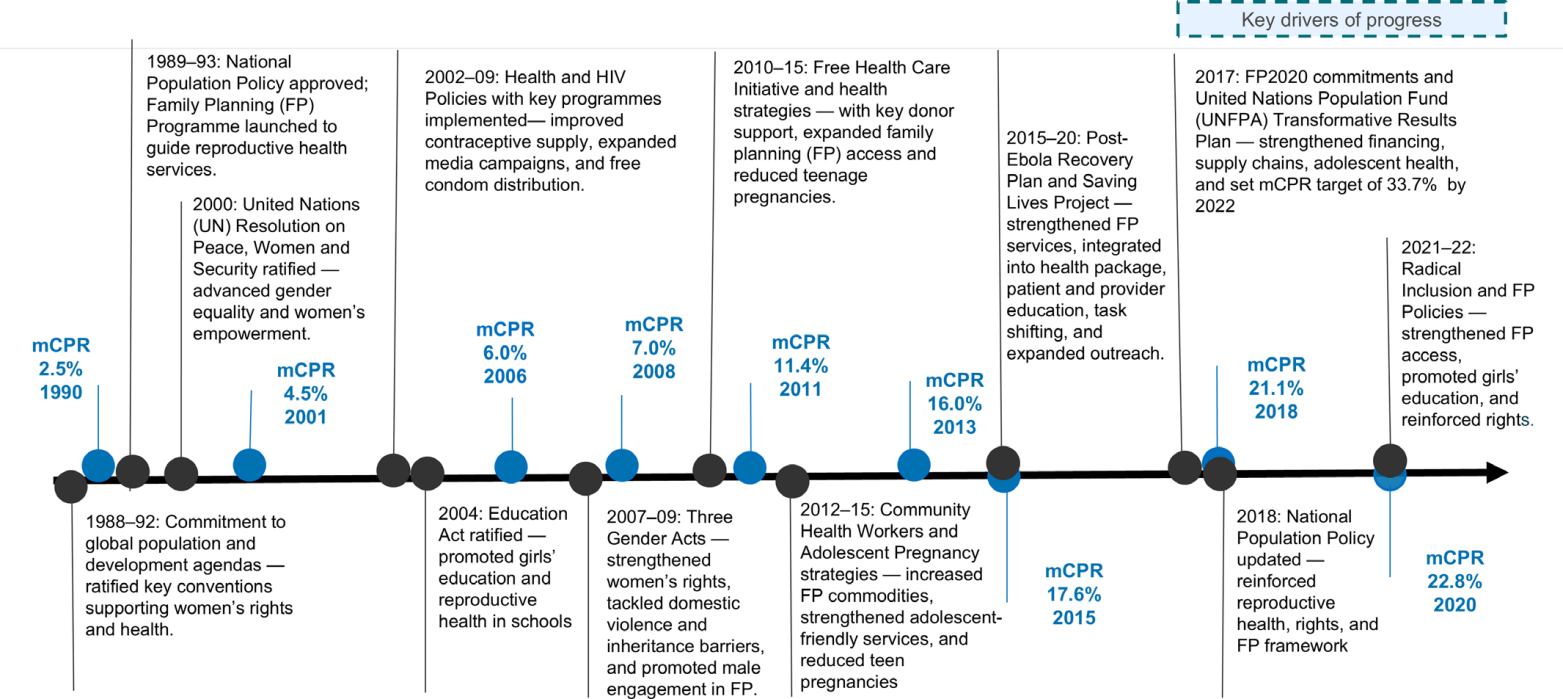
Impact of health policies and programmes on modern contraceptive prevalence (mCPR) trends in Sierra Leone, 1988–2022. Source: Adapted from Exemplars in Global Health template; data and analysis by authors.

A national review of policy, programme and financing documents identified four eras in Sierra Leone’s family planning trajectory (1990–2022), each marked by milestones in government action, donor support and service reform. Mapping these initiatives to mCPR trends (figure 1) showed that each policy or programme bundle preceded measurable mCPR gains. Figure 1 shows the timeline of major policies, programmes and changes in mCPR. mCPR estimates for married/in-union women are drawn from DHS surveys (2008, 2013 2019)^26^,^28^ and from UN modelled estimates for other years.^25^ Table 3 summarises how these policy and programme bundles map onto the study’s conceptual framework, with full details provided in online supplemental file 6.

**Table 3:** Mapping pathways from family planning policy and programme bundles (1990–2022) to study conceptual framework domains and modern contraceptive prevalence rate (mCPR) gains in Sierra Leone

Name of policy/ programme bundle	National commitment and institutional environment	Family planning financing, policy and programmes	Subnational/local context	Women’s outcomes
Foundation and Wartime Resilience (1990–2001)	The government formally prioritised reproductive health and family planning aligned national policy structures with global commitments, and established coordinating units despite conflict.	Supply chain established as a priority, Non-governmental organisations (NGOs) notably Marie Stopes pioneered the expansion of family planning services with sustained support from UNFPA and other donors, to reach diverse and conflict affected communities leading to the first national communication campaigns and improve awareness.	Outreach initiatives extended services to peri-urban areas, engaging community leaders and adapting strategies for conflict-affected populations through local radio and peer educators.	Early adopters, including displaced women, gained knowledge and began using contraceptives. Initial agency was evident among some women, and early shifts in social norms around family planning occurred.
Post-War Gender Empowerment (2002–2007)	After the civil war, national recovery focused on gender equity and child protection, institutionalised through legal reforms and policy integration with guidance from international frameworks.	Donor investments from UNFPA, FCDO, and the World Bank enabled a major scale-up of reproductive health and integrated HIV/family planning initiatives, with a focus on outreach, school-based education, and provider training.	Decentralisation improved local service delivery, and education reforms increased awareness. Community-based HIV/AIDS committees and livelihood support helped women and former combatants participate.	Social marketing and peer education expanded awareness and acceptance of family planning. Legal reforms delayed marriage and increased girls’ schooling, supporting women’s autonomy.
Fee Removal and Systems Strengthening (2008–2014)	The introduction of the Free Health Care Initiative and sector-wide reforms signalled high-level political commitment to maternal and reproductive health, integrating family planning into essential health services.	Large-scale pooled financing by UNFPA, FCDO and partners, along with supply chain reforms and performance-based incentives, improved continuous access to commodities and services, particularly for young people.	Expanded networks of peripheral health units and community health workers ensured access in rural and marginalised areas. Outreach increased through school programmes and mass communication. NGOs collaborated with government to scale rural outreach, youth services, and health worker support.	Women and adolescents gained reliable access to family planning, experienced increased knowledge and empowerment, and exercised greater autonomy in reproductive choices, with significant rises in modern contraceptive uptake.
Equity and Adolescent Innovations (2015–2023)	Equity and adolescent-focused reforms were institutionalised through digital monitoring, policy integration, and ambitious international targets, with a legal framework promoting girls’ education and inclusion.	Donor-funded supply chain and digital tracking initiatives (UNFPA, FCDO, WHO, UNICEF) supported adolescent-friendly, last-mile delivery models and institutionalised comprehensive sexuality education.	School-led and peer-led programmes expanded community and rural access. Mobile outreach and digital engagement targeted underserved youth.	Women and girls experienced enhanced agency, legal empowerment, improved access, and the highest ever satisfaction of family planning demand, resulting in record modern contraceptive prevalence.

Note: The column ‘Women’s outcomes’ encompasses both individual-level changes (such as knowledge, attitudes and uptake) and shifts in agency, autonomy and empowerment. For an expanded description of intermediate factors, see online supplemental Table S3.

UNICEF, United Nations Children’s Fund.World Bank, The World Bank Group; FCDO, UK’s Foreign, Commonwealth & Development Office; FCDO, UK’s Department for International Development; NGO, non-governmental organisation; UNFPA, United Nations Population Fund.

#### Foundation and wartime resilience (1990–2001)

National commitment to family planning, reinforced by UN technical support and advocacy for women’s health rights, led to a dedicated unit in 1992. Service delivery remained largely urban, donor-funded and NGO-led (Planned Parenthood Association of Sierra Leone (PPASL), Marie Stopes Sierra Leone), persisting throughout the war laying the foundation for post-war expansion.^30^,^39^

#### Post-war reconstruction & gender empowerment (2002–2007)

 Post-war policies and external financing strengthened the health system, prioritised adolescent and maternal health, and advanced gender equity legislation—including free and compulsory primary education and laws protecting against child marriage—broadening access and empowering women.^40^,^51^

#### Fee-removal and systems strengthening (2008–2013)

 The removal of user fees through the FHCI reduced financial barriers to service utilisation. This, together with strengthened supply chains and expanded training of health workers, improved contraceptive access, and quality. Embedding family planning within essential health services, supported by rights-based policies, ensured equitable provision. Growing media engagement and community outreach then raised awareness and demand, collectively contributing to accelerated mCPR gains.^52^,^63^

#### Equity, adolescent focus and last mile delivery (2014–2022)

 This period covers the Ebola response and post-Ebola recovery. Efforts during this period prioritised health system recovery and resilience, consistent commodity availability, and last-mile delivery, client and provider counselling and health education, expanding adolescent-friendly services and girls’ education through policies such as the Radical Inclusion Policy to promote equity, particularly for girls and rural communities. Together, these measures contributed to broader access and sustained gains in modern contraceptive use across Sierra Leone.^64^,^75^

### Thematic drivers of progress

#### Political commitment and advocacy

Sustained political will was manifested through landmark policies: the Education Act (2004), the most recent Radical Inclusion Policy (2021), Gender Acts (2007–2009)^45^,^47^ and FHCI (2010).^62 63^ Commitment was reinforced by alignment with global frameworks, such as the International Conference on Population and Development (ICPD), the Sustainable Development Goals (SDGs) and the Family Planning 2020/2030 initiatives (FP2020/FP2030), driving external investment and inclusion of family planning in national strategies.^70^ These reforms attracted donor support, strengthened women’s legal rights and expanded access.

#### External financing and partnerships

Strong donor-government alignment was essential throughout all eras. External financing from UNFPA, Global Fund, UK’s Foreign, Commonwealth & Development Office (FCDO) and others supported supply chain upgrades, workforce training and community mobilisation.^4348 51^,^56 59 61 65 67 72^ Cross-sector partnerships with NGOs and UN agencies facilitated integration of family planning into broader health and development agendas.^54^

#### Supply chain and service delivery

Reform highlights include the creation of the National Medical Supplies Agency (NMSA),^65^ improved procurement and distribution and expansion of primary health units providing long-acting reversible contraception. Integration of family planning with postpartum, post-abortion and immunisation services, plus widespread workforce training, improved access, particularly for adolescents and hard-to-reach populations.^54 57 65 67^

#### Community engagement and gender equality

Legal gains and active advocacy from chiefdom advocacy groups, male peer educators and national campaigns promoted gender equality, reproductive rights and delayed marriage. Programmes targeted stigma, promoted informed choice and integrated gender-based violence prevention into family planning services.^3640 41 45^,^47 49 50 58 60 74 75^

#### Demand generation

Mass media campaigns, behavioural change communication (BCC), school-based education, peer outreach and the introduction of comprehensive sexuality education underpinned demand generation.^60 69 72 76^ Such approaches increased knowledge and acceptance, reduced misconceptions and contributed to sustained mCPR growth. Decomposition analysis confirmed that knowledge, education and fieldworker outreach accounted for much of the increase in mCPR among women and adolescents. Details appear in online supplemental file 2.

## Qualitative analysis

### Participants

A total of 33 KIIs were conducted with family planning professionals (n=18) and community members (n=15) of whom adolescents (n=6) and adults (n=9). 12 FGDs were conducted with adolescent girls (n=2), boys (n=2), married women (n=2) married men (n=2), community influencers (n=2) and peripheral health unit (PHU) staff (n=2).

The themes that emerged from the qualitative analysis are discussed below:

#### Political commitment, policy and advocacy

The impact of policy reforms and political commitment on family planning was substantiated by qualitative analysis, with stakeholders emphasising the transformative effect of key policies:

One NGO director noted: “Free Health Care Initiative stands out, for me, as the most significant …and influential policy promoting family planning uptake.” (NGO Director)

One former Director at the Ministry of Health and Sanitation (MOHS) shared,

“The Free Health Care …has made a huge impact in terms of the provision of free family planning services in public facilities, so it’s quite an important policy.” (Formal Director, MOHS)

#### External financing and cross-sectoral partnerships

Evidence from key informant interviews demonstrated that collaboration among multiple stakeholders was central to mCPR growth. Stakeholders highlighted national and district steering committees and task forces as critical platforms for coordinating ministries, partners and community leaders in decision-making and information-sharing. As one participant noted:

“A lot of task forces and steering committees have been set up where you have representatives from the other ministries. Now traditional leaders are critical. You can find them in most of the steering committees of the Ministry of Health and Sanitation.” (Former Director, MOHS)

NGOs such as Marie Stopes Society of Sierra Leone (MSSL) and the PPASL, together with civil society groups, played pivotal roles not only in outreach and service delivery but also in policy advocacy. As one partner observed:

“We are also very much involved in policy dialogue. We will look at certain policies and tell them these policies are outdated. We need to review them. These all contributed to the policy shift in family planning.” (NGO Director)

#### Supply chain and service delivery

Qualitative interviews confirmed the effectiveness of supply chain reforms and expanded service delivery. A development partner stressed the significant role of UNFPA-led supply chains while also noting contributions from other donors:

“In terms of family planning products, UNFPA is the body that is bringing in family planning products…in terms of the funders for that, FCDO provide support in procuring the product.” (NGO Director)

Both community and stakeholder perspectives underscored service delivery gains: one highlighted improved availability at facilities and through outreach, while another pointed to the role of task-shifting and workforce scale-up in extending access to remote areas:

“We get Depo from the clinic, but Marie Stopes too provides that. We receive the emergency pills both at the clinic and from Marie Stopes when they come here.” (Married Woman, Matotoka)

“They started training community health workers because we believe that we needed to get to the hard-to-reach areas. So, government has over 15,000 community health workers.” (NGO Director)

#### Community engagement on gender equality and women’s reproductive rights

Qualitative findings reaffirmed the influence of community-based behaviour change and shifting gender norms. Community and religious leaders visibly championed gender equality enforced gender-based violence (GBV) bylaws and promoted women’s empowerment. This gradual shift was captured by an adolescent girl from Port Loko, who said:

“With the coming of this 50-50 initiative, women also see themselves being equal in decision-making like men.” (Adolescent Girl, Port Loko)

These qualitative perspectives highlighted the role of laws, advocacy, peer networks and social mobilisation in strengthening women’s reproductive agency and equality in communities.

#### Demand generation

Qualitative interviews demonstrated the practical impact of demand-generation strategies. Informants stressed the vital role of BCC in clinics and community outreach.

“In every clinic we have health education on family planning. So, family planning has become an educational tool within the services provided for our pregnant women and our lactating mothers.” (MOHS Medical Officer)

Community-based mobilisation and peer educator activity addressed myths and sociocultural barriers, while boosting engagement:

“There is now significant emphasis on the girl child, highlighting the importance of education for young girls. Some interventions have been implemented, which, directly or indirectly, impact the reproductive and sexual health of young girls.” (Former Director MOHS)

These perspectives showed the reach of advocacy and adolescent programmes, and the transformative effect of linking family planning with educational empowerment across society.

## Discussion

**Figure 2:**
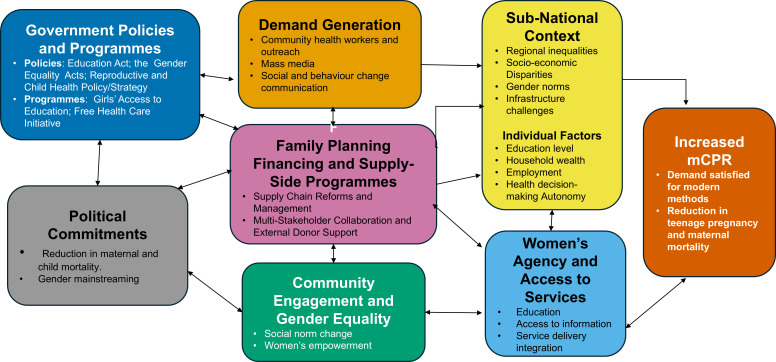
Theory of change illustrating drivers of modern contraceptive prevalence in Sierra Leone. mCPR, modern contraceptive prevalence rate.

This mixed-methods study analysed Sierra Leone’s rapid gains in mCPR. The drivers of progress identified in this study reflect the interplay of policy reforms implemented across four eras of Sierra Leone’s family planning journey—foundation and wartime resilience, post-war reconstruction and gender empowerment, fee removal and systems strengthening, and equity-focused last-mile delivery. Findings aligned with the study’s conceptual framework (figure 2) and demonstrated that each policy bundle contributed specific mechanisms and pathways; notably, the era of the FHCI marked a pivotal point, magnifying the impact of preceding reforms and propelling modern contraceptive uptake to new heights. Progress was driven by three core, mutually reinforcing factors: (1) high-level political commitment, most visible in the FHCI; (2) donor–government alignment and partnerships; and (3) community-based demand generation rooted in gender empowerment and expanded girls’ education.

Political commitment, embodied in the FHCI, created an enabling policy and institutional environment. High-level leadership, particularly from the President and First Lady,^62^ positioned maternal and child health and repositioned family planning as national priorities,^69 71^ with the FHCI widely regarded as an impactful flagship reform.^4 77^ The initiative strengthened the health workforce, improved supply chains and introduced greater accountability in resource use. This visibility mobilised domestic and external resources and spurred policy changes that reinforced service delivery and monitoring.^64^ By explicitly linking reproductive health to gender equality, the FHCI expanded service coverage and helped normalise family planning as a rights-based intervention.

In a constrained fiscal environment, close alignment with international partners became essential. Non-binding agreements such as the 2011 Country Compact fostered pooled resources, harmonised activities and joint monitoring.^55^ Major programmes, including FCDO’s Saving Lives and UNFPA’s Global Programme on Reproductive Health Commodity Security,^54 65^ provided funding, expertise and supply chain innovations such as electronic logistics systems and last-mile delivery.^65^ NGOs further extended outreach, particularly in underserved areas. Joint government, donor and civil society engagement also supported communication campaigns and peer-led advocacy that strengthened awareness, demand and uptake.^10 11^ Despite these achievements, dependence on donor financing represents a structural vulnerability, highlighting the importance of mobilising sustainable domestic resources.^78^

The FHCI and donor partnerships also accelerated gains in gender equality and women’s agency. Legislative reforms, including the Gender Acts, Education Act, and Sexual Offences Act, together with international commitments, created a supportive policy environment.^45^,^4771 75^ These were translated into empowerment programmes, adolescent-focused initiatives and advocacy opportunities. Community campaigns such as Hands Off Our Girls and Girls’ Access to Education^74 77^ mobilised leaders to end child marriage and reduce teenage pregnancy, while male peer networks promoted positive masculinity and shared responsibility for reproductive health.^54 56^ Comprehensive sexuality education was introduced in schools and extended to out-of-school youth, expanding knowledge and agency.^72^ Evidence from both qualitative and quantitative data demonstrated that female education and empowerment were linked to increased girls’ school retention, delayed marriage, contraceptive awareness and uptake.^11^ Nevertheless, progress has been uneven; rural areas and adolescents continue to face barriers, underscoring the need for sustained, locally tailored outreach.^10 11^

The combined effect of these drivers created a virtuous cycle of reform. Political commitment catalysed policy and system change; donor partnerships provided financing and technical support; and community demand generation ensured services were used. None of these factors alone accounts for the scale of progress; rather, their combined effect was decisive in Sierra Leone’s mCPR gains.

Sierra Leone’s trajectory echoes experiences in similar fragile settings: in Ethiopia, community-driven programmes like the Health Extension Programme boosted maternal healthcare access in rural areas; in Rwanda, the HRH Programme exemplifies how political leadership and strategic donor alignment can rapidly build health workforce capacity.^17 18^ Transferable lessons include the importance of sustained political prioritisation of family planning, combining system investments with measures to overcome sociocultural barriers, and maintaining community-driven demand generation through gender-transformative approaches. These principles provide pathways for achieving FP2030 commitments and broader reproductive health objectives.

Despite strong progress, barriers remain. Regional disparities, gender inequities and persistent rural supply challenges continue to undermine equity.^10 11^ Reliance on donor funding presents risks in the face of volatile financing. Sustained domestic investment in supply chains and workforce retention will be essential to secure long-term programme stability.

Study limitations must also be considered. Quantitative analyses relied on secondary datasets such as DHS, and UN sources, which differ in definitions, schedules and potential confounding. The scarcity of peer-reviewed publications necessitated the use of grey literature, introducing the possibility of bias. Qualitative results, based on purposive sampling, are subject to selection and social desirability bias, which may limit generalisability. Moreover, mCPR as the primary outcome captures only part of reproductive autonomy, as it does not fully measure unmet need, demand satisfied, or the complexity of women’s intentions. Interpretations must therefore remain cautious and context-specific.

## Conclusion and programmatic implications

This study provides practical guidance for policymakers and practitioners in Sierra Leone and other resource-constrained settings. The country’s substantial rise in mCPR illustrates the effectiveness of sustained political leadership and strategic partnerships. High-level commitment, reinforced by donor and civil society collaboration, enabled progress. Future initiatives should continue embedding family planning within broader health, education and gender strategies to ensure sustainability.

Key recommendations include integrating policies and accountability frameworks that link domestic and external actors; investing in distribution systems and health worker capacity; and prioritising community-driven demand generation, especially gender-transformative and youth-inclusive approaches. For Sierra Leone specifically, priorities should focus on building resilient, domestically funded supply chains; expanding youth-friendly, culturally adapted family planning services with targeted outreach; engaging men and boys as partners; and addressing myths, stigma and misinformation.

Ongoing donor dependency, inconsistent commodity supply (notably long-acting methods), sociocultural barriers and misinformation all present continuing challenges. Addressing these risks will be critical to ensuring that family planning programmes in Sierra Leone—and in similar high-fertility, resource-limited contexts—achieve both sustainability and equity.

## Supplementary material

10.1136/bmjgh-2024-018775 online supplemental figure 1

10.1136/bmjgh-2024-018775 online supplemental file 1

10.1136/bmjgh-2024-018775 online supplemental file 2

10.1136/bmjgh-2024-018775 online supplemental file 3

10.1136/bmjgh-2024-018775 online supplemental file 4

10.1136/bmjgh-2024-018775 online supplemental file 5

10.1136/bmjgh-2024-018775 online supplemental file 6

## Data Availability

Data are available upon reasonable request.
