# Supplementary figures and images for: Progress in family planning in Sierra Leone: a mixed-methods case study
Source: BMJ Glob Health. 2026 Jun 9;11(Suppl 3):e018775. doi: 10.1136/bmjgh-2024-018775 (PMC13250227; doi:10.1136/bmjgh-2024-018775)

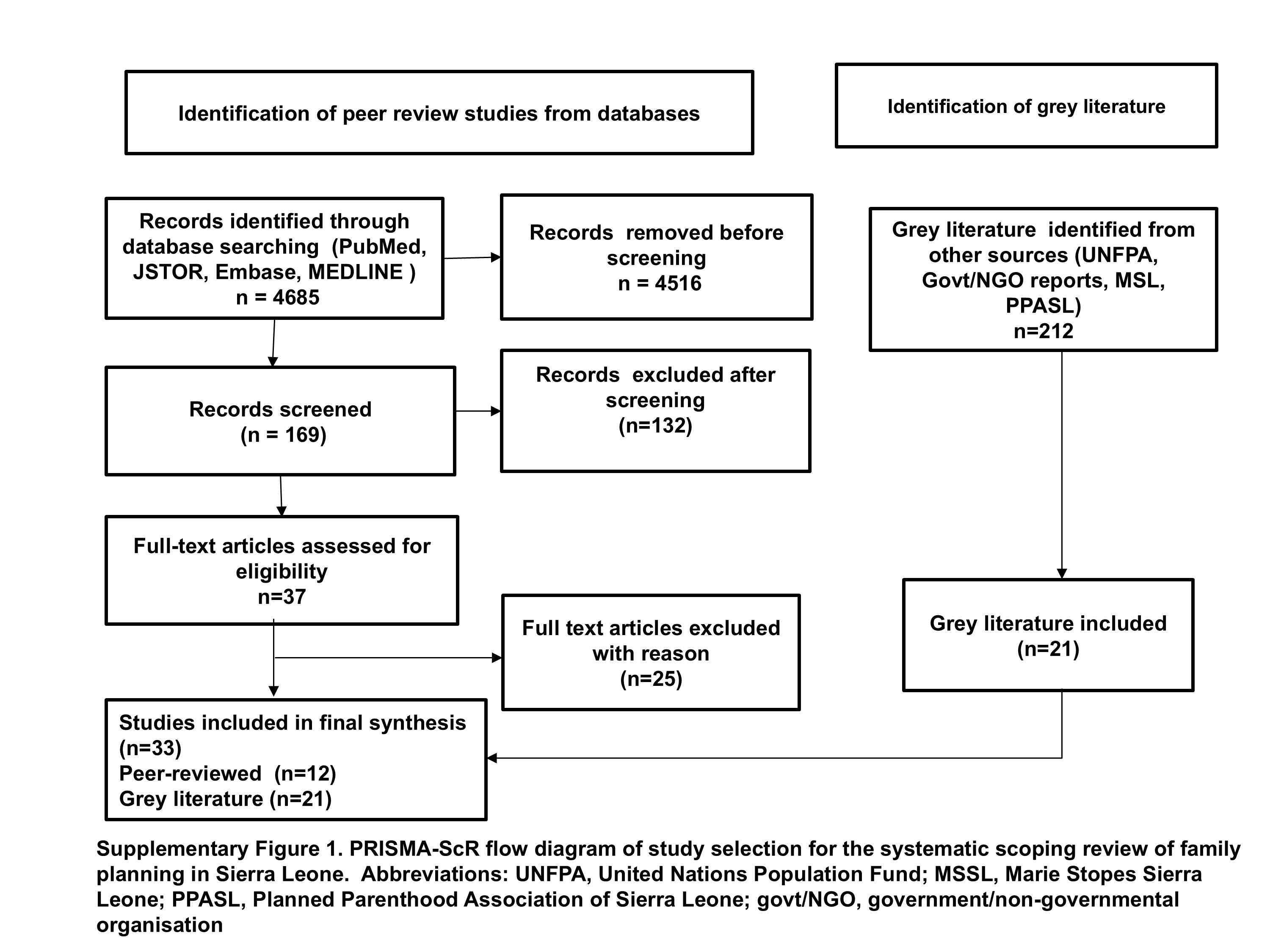

Supplement: online supplemental figure 1 [file bmjgh-11-Suppl_3-s001.tif]
