# Supplementary material for: Progress in family planning in Sierra Leone: a mixed-methods case study
Source: BMJ Glob Health. 2026 Jun 9;11(Suppl 3):e018775. doi: 10.1136/bmjgh-2024-018775 (PMC13250227; doi:10.1136/bmjgh-2024-018775)
Supplement: online supplemental file 1 [file bmjgh-11-Suppl_3-s002.docx]

Supplementary File 1. Search strategies for country-specific systematic scoping reviews: Sierra Leone case study

***Medline***

1. exp Contraceptive Agents/

2. exp Family Planning Services/

3. exp Contraception/

4. exp Reproductive Health Services/

5. contraception behavior/

6. (Contraceptive* or contraception or “birth control” or “vaginal ring*” or “intrauterine device*” or IUD or IUDs or IUCD or IUCDs or condom* or “female sterilization” or “female sterilization” or “male sterilization” or “male sterilization” or “vaginal barrier*” or diaphragm* or “cervical cap*” or spermidic* or “family planning service*” or vasectom* or “contraceptive injection*” or “contraceptive implant*” or “contraceptive injectable*”).tw,kf.

7. 1 or 2 or 3 or 4 or 5 or 6

8. (“Contraceptive prevalence rate” or “modern contraceptive prevalence rate” or mCPR or “contraception behavi*” or “demand satisfied” or “unmet need”).tw,kf.

9. (CPR not resuscit*).tw,kf.

10. 8 or 9

11. *Country X*/ or (*Country X* or *add other related terms e.g., regions*).tw,kf.

12. 7 or 10

13. 11 and 12

14. limit to 1990-current

Number of Hits=

***EMBASE***

1. exp vagina contraception/ or exp long-acting reversible contraception/ or exp hormonal contraception/ or exp barrier contraception/ or exp emergency contraception/ or exp contraception/ or exp oral contraception/ or exp family planning/ or exp contraceptive behaviour/ or exp contraceptive agent/

2. (contraception or “oral contraceptive pill*” or “contraceptive implant*” or “contraceptive injectable*” or “contraceptive injection” or “contraceptive patch*” or “vaginal ring*” or “intrauterine device*” or IUD or IUCD or “female condom*” or “male condom*” or “female sterilization” or “male sterilization” or “vaginal barrier” or diaphragm or “cervical cap*” or “spermicidal agent*” or “long acting reversible contraceptive*” or “contraceptive knowledge” or “contraceptive strateg*” or “hormonal contraceptive*” or “family planning service*” or “birth control” or “emergency contraceptive*” or “contraceptive agent*” or “reproductive health service*”).tw,kw.

3. (“Contraceptive prevalence rate*” or “modern contraceptive prevalence rate*” or mCPR or “contraception behavi*” or “demand satisfied” or “unmet need”).tw,kw.

4. (CPR not cardiopulmonary resuscitation).tw,kw

5. *Country X*/ or (*Country X* or *add other related terms e.g., regions*).tw,kw.

6. 1 or 2

7.3 or 4

8. 6 or 7

9. 5 and 8

10. Limit to 1990 to current

Number of hits=

***APA PsycInfo***

1. exp Contraceptive Devices/ or exp Birth Control/ or exp Oral Contraceptives/ or exp Condoms/ or exp Family Planning/ or exp family planning attitudes/
2. (contraception or “oral contraceptive pill*” or “contraceptive implant*” or “contraceptive injectable*” or “contraceptive injection*” or “contraceptive patch*” or “vaginal ring*” or “intrauterine device*” or IUD or IUCD or “female condom*” or “male condom*” or “female sterilization” or “male sterilization” or “vaginal barrier” or diaphragm or “cervical cap” or “spermicidal agent*” or “long acting reversible contraceptive*” or “contraceptive knowledge” or “contraceptive strateg*” or “hormonal contraceptive*” or “family planning service*” or “birth control” or “emergency contraceptive*” or “contraceptive agent*”or “reproductive health service*”).mp.
3. (“contraceptive prevalence rate” or “modern contraceptive prevalence rate” or mCPR or “contraception behavi*” or “demand satisfied” or “unmet need”).mp.
4. (CPR not cardiopulmonary resuscitation).mp.
5. *Country X*/ or (*Country X* or *add other related terms e.g., regions*).mp
6. 1 or 2
7. 3 or 4
8. 6 or 7
9. 5 and 8
10. Limit to 1990-current

Number of hits=

***CINAHL***

1. AB (MH "Contraceptives, Postcoital") OR (MH "Contraceptives, Oral") OR (MH "Long-Acting Reversible Contraceptives") OR (MH "Contraceptive Agents, Hormonal") OR (MH "Family Planning: Contraception (Iowa NIC)") OR (MH "Diaphragms, Contraceptive") OR (MH "Contraception Care (Saba CCC)")
2. AB ( (Contraceptive# or contraception or "birth control" or "vaginal ring#" or "intrauterine device#" or IUD or IUDs or IUCD or IUCDs or condom# or "female sterilisation" or "female sterilization" or "male sterilisation" or "male sterilization" or "vaginal barrier#" or diaphragm# or "cervical cap#" or spermidic* or "family planning service#" or vasectom* or “contraceptive injection#” or “contraceptive implant#” or “contraceptive injectable#”))
3. S1 OR S2
4. AB “Contraceptive prevalence rate” or “modern contraceptive prevalence rate” or mCPR or “contraception behavior” or “demand satisfied” or “CPR not cardiopulmonary resuscitation” or “unmet need”
5. AB (*Country X* or *add other related terms e.g., regions*)
6. S3 or S4
7. S6 and S5
8. Limit 1990-2000

Number of hits=

***Web of science***

1. “Contraceptive” or “contraception” or "birth control" or "vaginal ring” or "intrauterine device” or “IUD” or “IUDs” or “IUCD” or “IUCDs” or “condom” or "female sterilisation" or "female sterilization" or "male sterilisation" or "male sterilization" or "vaginal barrier" or “diaphragm” or "cervical cap" or “spermicide” or "family planning service" or “vasectomy “or “contraceptive injection” or “contraceptive implant” or “contraceptive injectable”
2. “Contraceptive prevalence rate” or “modern contraceptive prevalence rate” or” mCPR” or “contraception behavior” or “demand satisfied” or “CPR not cardiopulmonary resuscitation” or “unmet need”
3. “*Country X*” or *add other related terms*

Number of hits=

***JSTOR***

1. Contracepti*

AND

2. “Modern contraceptive prevalence rate” OR “demand satisfied” OR “unmet need”

AND

1. *Country X*

Number of hits=
