# Supplementary material for: Progress in family planning in Sierra Leone: a mixed-methods case study
Source: BMJ Glob Health. 2026 Jun 9;11(Suppl 3):e018775. doi: 10.1136/bmjgh-2024-018775 (PMC13250227; doi:10.1136/bmjgh-2024-018775)
Supplement: online supplemental file 2 [file bmjgh-11-Suppl_3-s003.docx]

Supplemental File 2. Operationalization of Key Family Planning Variables in Included Studies: Sierra Leone Scoping Review

| **Study ID (Author, Year)** | **Data source(s)** | **Key family planning variable** | **Data operationalization (i.e., how was the variable defined)** |
| --- | --- | --- | --- |
| Agbadi et al., 2020 | 2013 SLDH & SDHS data set. | Current use of modern contraceptive | Yes/No (using folkloric method, traditional method, and no method) |
| **Examples:** |  |  |  |
| Ali at al., 2018 | SARA survey 2010 & 2016 | Service availability | Mean score of 3 categories of facilities type (physical presence, personnel, and service utilization). The SARA survey assesses service availability as encompassing physical presence of delivery facilities, personnel, and service utilization. An average of the three components is used to assess this indicator. |
|  | SARA survey 2010 & 2016 | Readiness of health facilities to provide any of the following methods of contraception for unmarried adolescents | Oral contraceptive pills (COCS), male condoms, emergency contraceptive pills, and IUDs. |
|  | SARA survey 2010 & 2016 | Providing oral contraceptives in service. | Method choice and stock-out of oral contraceptive, injectable, and condoms |
| Amin, 1998 | 1993 Household Survey | Use of contraceptive | Use/not use |
|  | 1993 Household Survey | No of living children | <3, and > 3 children |
|  | 1993 Household Survey | Religion | Islamic or traditional religion |
|  | 1993 Household Survey | Education of respondent | No education or 1+ yrs. of education |
|  | 1993 Household Survey | Concrete house type | Concrete / not concrete |
|  | 1993 Household Survey | Modern items owned | Less than (<2 items) / More than (>2 items) |
| Amin, Chowdhury, Hill (1992) | 1990 Household Survey Data | Contraceptive use |  |
|  | 1990 Household Survey Data | Religion | Islam, Catholic, non-Catholic |
|  | 1990 Household Survey Data | Housing | Concrete/non-concrete |
|  | 1990 Household Survey Data | Level of education | Less than primary/higher than primary |
| Bongaarts & Hardee, 2019 | DHS | Prevalence (use) of modern contraception | Operationalization not specified but presumed (Yes/No) |
|  | DHS | Presence of Govt. FP program | Scores used to assess this performance ranged from 0-100 (0 = absence of FP program) to 100 (Theoretical strongest programs). This score was used to assess the government’s public FP programs. This public-sector program impact score was developed by the authors for use in another study. If no programs exist, a score as low as “0” could be assigned. However, programs perceived to be of high quality and broad scope of services could be scored up to 100. |
|  |  | GNI per capita | Operationalization not specified |
|  |  | Percent of pop that is urban | Not specified |
| Cahill et al., 2018 | Family Planning Estimation Tool to construct estimates & FPET (historical survey data). | mCPR- | % of women or partner who reported using at least one method of contraception |
|  |  | Unmet need | % of women who want to stop or delay childbearing but not currently using any method to prevent pregnancy |
|  |  | Demand satisfied | % of women whose demand for contraception was satisfied with a modern method. |
| James et al., 2022 | Global School-based Student Health Survey (GSHS) datasets | Use of condoms, other birth control methods (other than a condoms), any birth control method at last sexual intercourse. | Yes, No, I don’t know |
| Koroma et al., 2021 | Local Survey Data (using Lot Quality Assurance Sampling | Use more modern contraception | Currently using any method – yes/no |
|  | Local Survey Data (using Lot Quality Assurance Sampling | Belief in effectiveness of traditional method | Modern vs. rope/herbs |
|  |  |  |  |
|  | Local Survey Data (using Lot Quality Assurance Sampling | Stockouts of contraceptives | Check mark – Increased vs dropped |
| Labat et al., 2018 | Household Quantitative Survey . This assessment was conducted in 2016 at the end of the Ebola outbreak, based on a representative sample of young persons (10 -24 yrs.) in Sierra Leone to monitor the implementation of a UNFPA package of interventions focusing on healthy lifestyle behaviors . It is a household quantitative survey with open-ended questions. | Use of condoms and/or contraception | Contraception = utilization of modern contraceptive commodities (Yes/No)  Condom or contraception use = dual protection, (ii) condom alone (iii) contraception alone |
|  |  | Distance to health facility | <30 mins  >30 mins |
|  |  | Negotiation (condom & refusal) | Neither option  One out of two options  Both options |
|  |  | Heard of FP methods | 0-3 times  4-8 times |
| Sarnak & Gemmill, 2022 | DHS | Contraceptive use | Covert use versus overt use of contraception.  DHS couple data were used to assess covert versus open use of contraceptives. |
| Sserwanja et al., 2022 | 2019 DHS | Age at first sex | Continuous |
|  | 2019 DHS | Exposure to FP messages through TV, newspapers, or radio | Participants were asked if exposed to FP messages on radio, television, newspaper/magazine, or mobile texts with Yes/No responses. Responses were entered in the model individually. |
|  | 2019 DHS | Utilization of any modern contraceptives | Binary = Yes was coded (1)/no (0) |
|  | 2019 DHS | Religion | Muslim/Christian and others |
|  | 2019 DHS | Residence | Rural versus urban |
|  | 2019 DHS | Level of education | No edu., Primary, Sec, tertiary |
|  | 2019 DHS | Region | North, East, South, West, Northwest |
|  | 2019 DHS | Having visited health facility within last 12 months | Yes/No |
|  | 2019 DHS | Having been visited by health worker within 12 mo | Yes/No |
| Sserwanja, Nuwabaine, Kamara et al. 2023 | 2019 SLDHS | Exposure to FP mass media messages | Yes/No |
|  | 2019 SLDHS | Source of FP method | Government vs private facilities |
|  | 2019 SLDHS | Residence | Rural vs. urban |
|  | 2019 SLDHS | Region | Northern, Eastern, Southern, Western, Northwestern |
|  | 2019 SLDHS | Religion | Muslim and Christian and others |
|  | 2019 SLDHS | Level of education | No edu, primary, secondary, and tertiary |
|  | 2019 SLDHS | Wealth index | Richest, richer, middle, poorer, and poorest quintiles |
|  | 2019 SLDHS | Having visited health clinic with last 12 months | Yes/No |
|  | 2019 SLDHS | Problems with distance to nearest health facility | No big problems and big problems |

**References (peer-reviewed literature)**

1. Ali M, Farron M, Dilip TR, Folz R. Assessment of family planning service availability and readiness in 10 African countries. *Glob Health Sci Pract*. 2018;6(3):473–83. doi:10.9745/GHSP-D-18-00041
2. Amin R. Contraceptive use and desire for more children in two rural districts of Sierra Leone. *J Biosoc Sci*. 1998;30(3):287–96. doi:10.1017/S0021932098002879
3. Amin R, Chowdhury J, Hill RB. Socioeconomic differentials in contraceptive use and desire for more children in Greater Freetown, Sierra Leone. *Int Fam Plan Perspect*. 1992;18(1):24–6.
4. Agbadi P, Tawiah T, Frempong AK, Owusu S. Predictors of current use of modern contraceptives among married or in-union women in Sierra Leone: insights from the 2013 Demographic and Health Survey. *PLoS One*. 2020;15(4):e0231630. doi:10.1371/journal.pone.0231630 *(Also cited in the main manuscript reference list.).*
5. Bongaarts J, Hardee K. Trends in contraceptive prevalence in sub-Saharan Africa: the roles of family planning programs and education. *Afr J Reprod Health*. 2019;23(3):96–105. doi:10.29063/ajrh2019/v23i3.*9 (Also cited in the main manuscript reference list.).*
6. Cahill N, Sonneveldt E, Stover J, Weinberger M, Williamson J, Wei C, et al. Modern contraceptive use, unmet need, and demand satisfied among women of reproductive age who are married or in a union in the focus countries of the Family Planning 2020 initiative: a systematic analysis using the Family Planning Estimation Tool. *Lancet*. 2018;391(10123):870–82. doi:10.1016/S0140-6736(17)33104-5 *(Also cited in the main manuscript reference list.).*
7. James PB, Osborne A, Babawo LS, Bah AJ, Margao EK. The use of condoms and other birth control methods among sexually active school-going adolescents in nine sub-Saharan African countries. *BMC Public Health*. 2022;22:2358. doi:10.1186/s12889-022-14855-6 *(Also cited in the main manuscript reference list.).*
8. Koroma AS, Kamara HI, Moses F, Bah M, Turay M, Kandeh A, et al. Impact on key indicators of reproductive and child health after changes in program modalities in Sierra Leone, 2019. *Health Sci Rep*. 2021;4(2):e297. doi:10.1002/hsr2.297.
9. Labat A, Medina M, Elhassein M, Karim A, Jalloh MB, Dramaix M, et al. Contraception determinants in youths of Sierra Leone are largely behavioral. *Reprod Health*. 2018;15:66. doi:10.1186/s12978-018-0504-9 *(Also cited in the main manuscript reference list.).*
10. Sarnak DO, Gemmill A. Perceptions of partners’ fertility preferences and women’s covert contraceptive use in eight sub-Saharan African countries. *Stud Fam Plann*. 2022;53(3):583–600. doi:10.1111/sifp.12206
11. Sserwanja Q, Turimumahoro P, Nuwabaine L, Kamara K, Musaba M. Association between exposure to family planning messages on mass media and utilization of modern contraceptives among young women in Sierra Leone: insights from the 2019 Demographic and Health Survey. *BMC Womens Health*. 2022;22:376. doi:10.1186/s12905-022-01974-w *(Also cited in the main manuscript reference list.).*
12. *Sserwanja Q, Nuwabaine L, Kamara K, Musaba MW.* Determinants of quality contraceptive counselling information among young women in Sierra Leone: insights from the 2019 Sierra Leone Demographic and Health Survey. BMC Womens Health 2023;23:266. doi:10.1186/s12905-023-02419-8
