# Supplementary material for: Progress in family planning in Sierra Leone: a mixed-methods case study
Source: BMJ Glob Health. 2026 Jun 9;11(Suppl 3):e018775. doi: 10.1136/bmjgh-2024-018775 (PMC13250227; doi:10.1136/bmjgh-2024-018775)
Supplement: online supplemental file 3 [file bmjgh-11-Suppl_3-s004.docx]

Supplementary File 3: Grey literature sources on family planning and reproductive health in Sierra Leone

| **Study ID** | **Title** | **Authors/Agency** | **Year** | **Study Design** | **Participants** | **Data Source** | **Key Outcomes** | **Main Findings** |
| --- | --- | --- | --- | --- | --- | --- | --- | --- |
| 1 | Sierra Leone Demographic and Health Survey 2008 | Statistics Sierra Leone & ICF Macro | 2009 | National household survey | 7,374 women (15–49), 3,280 men (15–59) | DHS Final Report [link](https://dhsprogram.com/publications/publication-fr225-dhs-final-reports.cfm) | mCPR 8.2%; demand satisfied 22.9%; unmet need 27.6% | Low contraceptive use (6.7% married, 24.5% unmarried); urban–rural disparities; supported by GoSL, USAID, UNFPA, UNDP, UNICEF, DFID, WB. |
| 2 | Improving the SRH of uprooted communities (6 districts) | Marie Stopes Sierra Leone | 2011 | Program evaluation (EU project) | Six districts, later expanded | MSSL | mCPR rose from 8.2% (2008) to 14% (2009) | >1.1M contraceptives delivered; ~31,500 unintended pregnancies and 222 maternal deaths averted. |
| 3 | Survey of Availability of Modern Contraceptives (2011) | UNFPA Sierra Leone | 2011 | Health facility survey | 108 SDPs | UNFPA | >87% facilities offered ≥3 methods | Stockouts common; barriers include distance, demand, staffing. |
| 4 | Sierra Leone Demographic and Health Survey 2013 | Statistics Sierra Leone & ICF International | 2014 | National household survey | 16,658 women (15–49), 7,262 men (15–59) | DHS Final Report [link](https://dhsprogram.com/publications/publication-fr297-dhs-final-reports.cfm) | mCPR 20.9%; demand satisfied 40%; unmet need 25% | Significant rise in contraceptive use (15.6% married, 56.3% unmarried); 68% of users sourced FP from public facilities. |
| 5 | Annual Report 2015 – Resilient Growth | Marie Stopes Sierra Leone | 2015 | Annual program report | National | MSSL | CYPs: 360,160 in 2015 | Popular methods: pills (188k), injectables (107k), implants (47k); scale-up during Ebola outbreak. |
| 6 | IRMNH Programme Effectiveness Survey 2 | UNFPA | 2016 | Household survey (youth focus) | 214 respondents (12–45 yrs) | UNFPA Sierra Leone | mCPR 21.4%; unmet need substantial | 85% FP clients unable to read; youth-friendly service gaps; ~44% of females used health facility in past year. |
| 7 | Sierra Leone Multiple Indicator Cluster Survey 2017 | Statistics Sierra Leone | 2017 | National household survey | 17,873 women (15–49), 7,415 men | UNICEF/SSL report | Demand satisfied 56.7%; unmet need 27.7% | Marked increase in demand satisfied by modern FP methods. |
| 8 | FP2020 Commitment | Government of Sierra Leone | 2017 | Policy commitment | National | FP2020 site | mCPR 22.3% | Govt pledged to raise health budget and reduce adolescent unmet need. |
| 9 | FP2020 Country Action Plan | Government of Sierra Leone | 2017 | Strategic plan | National | FP2020 | mCPR target 33.7% by 2022 | Priorities: costed implementation plan, 10adolescent FP, supervision system. |
| 10 | RMNCAH Strategy 2017–2021 | MoHS | 2017 | National strategy | National | MoHS | mCPR baseline 23.6% | Linked to Agenda for Prosperity strategy. |
| 11 | SLFPCIP 2018–2022 | MoHS Sierra Leone | 2017 | Costed Implementation Plan | National | MoHS | mCPR baseline 24.1% | Target 33% by 2022; adolescent unmet need highest (30.7%). |
| 12 | National Health Facility Assessment on RH Commodities | UNFPA | 2019 | Cross-sectional health facility survey | National service delivery points | UNFPA | mCPR 20.4%; demand satisfied 45% | Frequent stockouts; service gaps linked to supply chain issues, staffing, demand. |
| 13 | Sierra Leone Demographic and Health Survey 2019 | Statistics Sierra Leone & ICF | 2020 | National household survey | 15,574 women (15–49), 7,197 men (15–59) | DHS Final Report [link](https://dhsprogram.com/publications/publication-FR365-DHS-Final-Reports.cfm) | mCPR 23.9%; demand satisfied 46.1%; unmet need 24.8% | Gradual increase in mCPR; unmet need decreased slightly; 95% of women knew ≥1 FP method. |
| 14 | Organisational Capacity Statement | Marie Stopes Sierra Leone | 2021 | Program report | National coverage | MSSL | mCPR >40% demand satisfied (2021) | 234,000 FP users (2021); 96,995 unsafe abortions averted; £9.2m costs saved. |
| 15 | FP2030 Indicator Summary Sheet: 2022 Measurement Report | Govt. of Sierra Leone, Track20 | 2022 | Estimates from FPET | Women 15–49 | FP2030 [link](https://fp2030.org/) | mCPR increased from 17.4% (2012) to 26% (2022); demand satisfied rose from 43% to 55%; unmet need declined from 23.1% to 20.8% | ~210,000 unsafe abortions averted; ~1,700 maternal deaths prevented by 2022. |
| 16 | Client Exit Survey (CES) 2022 | Marie Stopes Sierra Leone | 2022 | Facility exit survey | 361 clients (13–49 yrs) | MSSL HQ | 51% implants; 31% injectables; 12% pills | 99% satisfaction with FP services; high uptake among young women and students; 36% first-time users. |
| 17 | SLFPCIP 2023–2027 | MoHS Sierra Leone | 2022 | Costed Implementation Plan | National | MoHS | mCPR baseline 24.7% | Priorities: PPFP, stockout reduction, SBC. |
| 18 | National Family Planning Policy | MoHS Sierra Leone | 2022 | Policy document | National | MoHS | mCPR 26% | Priorities: quality services, access, SBC, financing, governance. |
| 19 | National Family Planning Guidelines | MoHS Sierra Leone | 2022 | Service delivery guidelines | National | MoHS | – | Standardizes FP provision across SDPs; aligns with WHO standards. |
| 20 | National Strategy for Reduction of Adolescent Pregnancy & Child Marriage 2018–2022 | Government of Sierra Leone | 2022 | Multisector strategy | Adolescents & young people | GoSL | – | Multisector commitments (MoHS, MSWGCA, MoYA, MBSSE, MLGRD); focus on CSE, AYF services, community empowerment. |
| 21 | UNFPA Sierra Leone overview | UNFPA | 2023 | Program summary | Women 15–49 | UNFPA website | mCPR 26%; unmet need ~20% | Provides national FP indicators (2022). |

**References (grey literature)**

1. Statistics Sierra Leone (SSL), ICF Macro. *Sierra Leone Demographic and Health Survey 2008*. Calverton (MD): SSL and ICF Macro; 2009.
2. Marie Stopes Sierra Leone (MSSL). *Improving the sexual reproductive health of uprooted communities in six districts of Sierra Leone*. Freetown: MSSL; 2011.
3. UNFPA Sierra Leone. *Survey of availability of modern contraceptives and essential life-saving maternal and reproductive health medicines in service delivery points in Sierra Leone: analytical report*. Freetown: UNFPA; 2011.
4. Statistics Sierra Leone (Stats SL), ICF International. *Sierra Leone Demographic and Health Survey 2013*. Freetown, Sierra Leone and Rockville (MD): Stats SL and ICF; 2014.
5. Marie Stopes Sierra Leone (MSSL). *A year of resilient growth: 2015 annual report*. Freetown: MSSL; 2015.
6. UNFPA. *Final report survey 2: multi-year annual survey to monitor programme effectiveness of the “Improving Reproductive Maternal and Newborn Health (IRMNH) Programme”*. Freetown: UNFPA; 2016.
7. Statistics Sierra Leone. *Sierra Leone Multiple Indicator Cluster Survey 2017: survey findings report*. Freetown: SSL; 2018.
8. Government of Sierra Leone. *Family Planning 2020 Commitment*. Freetown: GoSL; 2017.
9. Government of Sierra Leone. *Family Planning 2020 Country Action: opportunities, challenges and priorities*. Freetown: GoSL; 2017.
10. Ministry of Health and Sanitation (MoHS). *Sierra Leone RMNCAH Strategy 2017–2021*. Freetown: MoHS; 2017.
11. Ministry of Health and Sanitation (MoHS). *Sierra Leone Family Planning Costed Implementation Plan 2018–2022*. Freetown: MoHS; 2017.
12. UNFPA. *National health facility assessment on availability of reproductive health commodities and services in Sierra Leone*. Freetown: UNFPA; 2019.
13. Statistics Sierra Leone (Stats SL), ICF. *Sierra Leone Demographic and Health Survey 2019*. Freetown, Sierra Leone and Rockville (MD): Stats SL and ICF; 2020.
14. Marie Stopes Sierra Leone (MSSL). *Organisational capacity statement*. Freetown: MSSL; 2021.
15. Government of Sierra Leone. *FP2030 Indicator Summary Sheet: 2022 Measurement Report*. Track20; 2022.
16. Marie Stopes Sierra Leone (MSSL). *Client exit survey: final report*. Freetown: MSSL; 2022.
17. Ministry of Health and Sanitation (MoHS). *Sierra Leone Family Planning Costed Implementation Plan 2023–2027*. Freetown: MoHS; 2022.
18. Ministry of Health and Sanitation (MoHS). *National Family Planning Policy*. Freetown: MoHS; 2022.
19. Ministry of Health and Sanitation (MoHS). *National Family Planning Guidelines*. Freetown: MoHS; 2022.
20. Government of Sierra Leone. *National Strategy for the Reduction of Adolescent Pregnancy and Child Marriage 2018–2022*. Freetown: GoSL; 2022.
21. UNFPA. *Sierra Leone overview*. Freetown: UNFPA; 2023.
