# Supplementary material for: Progress in family planning in Sierra Leone: a mixed-methods case study
Source: BMJ Glob Health. 2026 Jun 9;11(Suppl 3):e018775. doi: 10.1136/bmjgh-2024-018775 (PMC13250227; doi:10.1136/bmjgh-2024-018775)
Supplement: online supplemental file 4 [file bmjgh-11-Suppl_3-s005.pdf]

Supplementary File IV: Detailed Oaxaca–Blinder Decomposition Analysis: Family Planning Trends in Sierra Leone

Prepared by the Center of Global Child Health-SickKids, October 2024

1 Methodology

1.1 Oaxaca-Blinder Decomposition

The SickKids (SK) team used the Oaxaca-Blinder decomposition method[1,2] to assess determinants of change in modern contraceptive prevalence rate (mCPR) in Sierra Leone using data from the 2008 and 2019 Demographic and Health Survey (DHS) waves. The analytic model was based on the conceptual framework (see below) created by the Family Planning Exemplars in Global Health (FP EGH) research consortium along with published literature on FP outcomes in low- and middle-income countries. Variables were measured at the individual level (i.e., for each observation) or at the ecological level (by using the regional mean or prevalence). Ecological-level variables were created if there were sufficient observations (n≥25 responses) in the respective district and/or if they were not collected for the entire evaluation sample due to the skip pattern. The evaluation

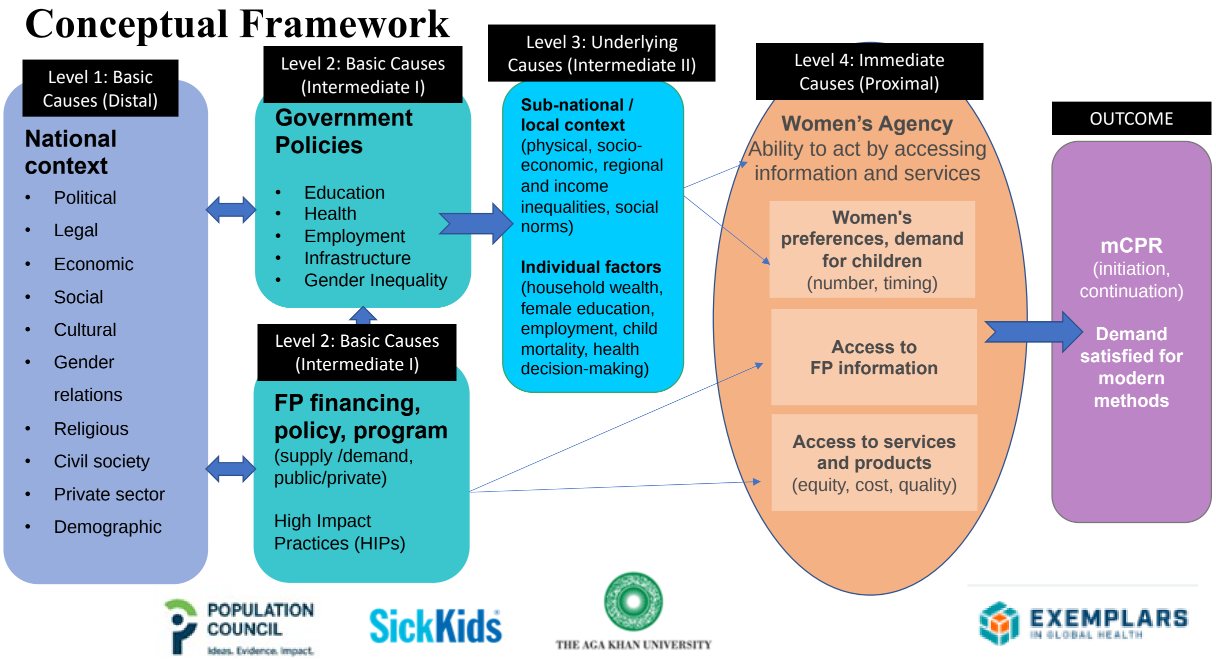

---

[1] JannB. The Blinder–Oaxaca decomposition for linear regression models.StataJ2008;8:453–79.

[2] Derek Headey, John Hoddinott, Seollee Park, Accounting for nutritional changes in six success stories: A regression-decomposition approach, Global Food Security, Volume 13, 2017, Pages 12-20,
ISSN 2211-9124, https://doi.org/10.1016/j.gfs.2017.02.003.
(https://www.sciencedirect.com/science/article/pii/S2211912416300992)

The models were developed by conducting bivariate analyses between mCPR and each independent variable (potential determinant/driver). Independent variables (IVs) with a p-value less than or equal to 0.25 were entered together into a linear probability model to determine the association between mCPR and IVs between the two time points. The IVs that were significant at a p-value less than or equal to 0.05, after adjusting for other covariates, were retained to be entered into the final model. In the final model, IVs with collinearity-values (VIF) >10 were dropped from the analysis. Province, religion, age, and year of survey were considered control variables. Districts were assigned to four provinces: Eastern (Kailahun, Kenema, Kono), Northern (Bombali, Kambia, Koinadugu, Port Loko, Tonkolili), Southern (Bo, Bonthe, Moyamba, Pujehun), and Western (Western area rural, Western area urban). The final estimates of change in mCPR explained by each determinant were calculated by multiplying the change (over time) in the IV by the respective beta-coefficient.

Women were analyzed by age group: (1) 15-49-olds, (2) 15-24-year-olds, and (3) 25-49-year-olds, and relationship status: (1) all women (regardless of relationship status), and (2) married/in-union women only, for a total of six models. Eligibility criteria for inclusion in the analyses were: had recent sex (within past 12 months), not currently pregnant, not in menopause, and fecund.

2 Results

2.1 Descriptives

2.1.1 All Women

Table 1. Descriptive Statistics of Women included in the All Women Analyses						
	15-24-year-olds		25-49-year-olds		15-49-year-olds	
**Survey year**	**2008**	**2019**	**2008**	**2019**	**2008**	**2019**
**Eligibility criteria**						
Had sex in past 12 months (%)	72.8	81.6	76.5	83.7	75.4	83
Not currently pregnant (%)	90	93.4	92.8	94	91.9	93.8
Not menopausal (%)	99.9	99.9	95.2	93.6	96.8	96.1
Fecund (%)	96.5	99.3	95.6	95.5	95.9	97
Total Eligible (N) - ***unweighted***	1,100	3,221	2,960	6,612	4,060	9,833
**Outcome: Current use of any modern contraceptive method (%)**	14.9	44.7	10.6	31.8	11.7	36
**Sociodemographic characteristics**						
Respondent's age (in years) (mean, SD)	19.9 (2.5)	19.9 (2.6)	33.8 (6.5)	34.5 (6.6)	30.2 (8.4)	29.7 (8.9)
Respondent's years of education (mean, SD)	4.1 (4.4)	7.3 (4.1)	2.2 (4.1)	3.5 (4.9)	2.7 (4.3)	4.7 (5.0)
Currently married/living with partner (%)	52.5	32	86.9	82.1	77.9	65.9
Wealth Index (1-10) (mean, SD)	5.5 (3.2)	5.9 (3.1)	5.0 (3.1)	5.6 (3.3)	5.1 (3.1)	5.7 (3.2)
Age at first sex (mean, SD)	15.4 (2.1)	15.7 (2.0)	15.8 (2.8)	15.9 (2.3)	15.7 (2.6)	15.8 (2.2)
Recent employment (%)	60.4	55.7	85.4	85.9	78.9	76.1
Urban residence (%)	45.2	54.5	34.5	44.9	37.3	48
**Family Planning and Health Systems Interventions**						
Source of family planning for users						
**Public** – gov't clinic/pharmacy, gov't home/community delivery (%)	48.8	79.9	59	82.5	55.9	81.4
**Private** - Private clinic/delivery, pharmacy (%)	51.2	20.1	41	17.5	44.1	18.6
Family Planning methods ever heard of						
Modern methods ever heard of (mean, SD)[1]	3.0 (2.4)	7.6 (2.4)	2.9 (2.5)	7.6 (2.5)	2.9 (2.4)	7.6 (2.4)
Contraceptive Pill (%)	65.8	97.5	63.5	97.3	64.1	97.4
Intrauterine Device (IUD) (%)	31.7	75.2	28.5	75.3	29.3	75.2
Injectables (%)	63.6	97.9	63.3	97	63.4	97.3
Implants (Norplant) (%)	4.4	97.7	4.2	96.8	4.3	97.1
Diaphragm (%)	NA	NA	NA	NA	NA	NA
Foam and Jelly (%)	4.9	NA	4.4	NA	4.5	NA
Male Condom (%)	67.9	96.8	59.7	95.7	61.8	96.1
Female Condom (%)	22.5	73.2	17.9	69	19.1	70.4
Female Sterilization (%)	20	64.2	25.3	66.6	23.9	65.9
Male Sterilization (%)	6	34.1	8	33.8	7.5	33.9
Emergency Contraception (%)	8.7	43.5	6.3	41.1	6.9	41.9
Lactational Amenorrhea Method (LAM) (%)	8.8	47.1	9.6	57.3	9.4	54
Standard Days Method (SDM)/Cycle Beads (%)	NA	32.2	NA	34.4	NA	33.7
Other Modern Method (%)	0	3.4	0	4.8	0	4.3
**Traditional Methods**						
Periodic Abstinence (Rhythm) (%)	13.9	39.9	11.7	40.8	12.3	40.5
Withdrawal (%)	16	63.5	12.6	59.8	13.5	61
Other Traditional Methods (%)	33.2	8.9	39	15.3	37.5	13.2

**Current modern methods used:**						
Contraceptive Pill (%)	42.2	13.4	39.6	22.4	40.4	18.8
IUD (%)	3.2	1.7	4.1	2	3.8	1.9
Injectables (%)	25.1	42	46.4	42.7	39.3	42.4
Implants (%)	0	40.3	0	30.1	0	34.2
Diaphragm (%)	NA	NA	NA	NA	NA	NA
Foam and Jelly (%)	NA	NA	NA	NA	NA	NA
Male condom (%)	26.6	1.8	7.1	0.7	13.6	1.1
Female condom (%)	0	0	0	0.05	0	0.03
Female sterilization (%)	0	0	0.7	0.9	0.5	0.6
Male sterilization (%)	0	0	0	0.1	0	0.08
Emergency Contraception (%)	NA	0.4	NA	0.7	NA	0.6
LAM (%)	3	0.2	2.1	0.1	2.4	0.1
SDM/Cycle Beads (%)	NA	0.2	NA	0.3	NA	0.2
**Reasons for contraceptive method discontinuation (%)**						
Became pregnant while using	NA	2.3	NA	1.8	NA	1.9
Wanted to become pregnant	NA	17.4	NA	28	NA	24.7
Husband/partner disapproved	NA	4.6	NA	3.8	NA	4.1
Side effects	NA	50.3	NA	45.1	NA	46.7
Health concerns	NA	0	NA	0	NA	0
Access, availability (lack of access/too far)	NA	1.7	NA	1.2	NA	1.3
Wanted more effective method	NA	5.9	NA	6.5	NA	6.3
Inconvenient to use	NA	5.7	NA	3.7	NA	4.3
Infrequent sex, husband away	NA	4.7	NA	5.1	NA	4.9
Cost too much	NA	2.4	NA	1.3	NA	1.6
Fatalistic	NA	0.2	NA	0.3	NA	0.3
Difficult to get pregnant/ menopausal	NA	0	NA	0.2	NA	0.2
Marital dissolution/Separation	NA	0	NA	0.5	NA	0.3
Other	NA	3.9	NA	2.4	NA	2.9
Don’t know	NA	0.9	NA	0.2	NA	0.4
**Contacts with healthcare**						
Visited by family planning worker in past 12 months (%)	8.7	27.2	8.6	2 9.3	8.6	28.6
Visited health facility in past 12 months (%)	33.2	48.3	37.1	55	36.1	52.8
Recent exposure to family planning messages in last 12 months						
On radio (%)	53	33.9	46.8	33	48.5	33.3
In newspaper (%)	9.8	2.8	6.1	2.9	7.1	2.9
On TV (%)	8.1	12.9	7.1	13.4	7.4	13.2
**Exposure to mass media (%)**						
Frequency of reading the newspaper						
not at all	80.3	88.8	89.4	92.4	87	91.2
< once a week	7.6	8.1	4.8	4.8	5.5	5.9
at least once a week	10.5	3.1	4.9	2.9	6.3	2.9
Almost everyday	1.6	NA	0.9	NA	1.1	NA
Frequency of listening to radio (%)						
not at all	38.1	51.4	42.8	52.7	41.6	52.3
< once a week	10.9	24	9.9	20.1	10.1	21.4
at least once a week	23.3	24.6	20.4	27.2	21.2	26.3
Almost everyday	27.7	NA	27	NA	27.2	NA
Frequency of watching television (%)						
not at all	75	66.7	85.9	71	83.1	69.6
< once a week	9.3	16.5	4.6	13.2	5.8	14.3
at least once a week	9.8	16.8	5.8	15.8	6.9	16.1

Almost everyday	6	**NA**	**3.7**	**NA**	**4.3**	**NA**
**Getting medical help for self (issues deemed a big problem):**						
Getting permission to go (%)	8.3	22.3	7	23.7	7.4	23.3
Getting money for treatment (%)	79.5	62.7	81	67	80.6	65.6
Distance to health facility (%)	49.7	37.9	54.8	44.2	53.4	42.1
Not wanting to go alone (%)	20.2	17.7	18.7	21	19.1	19.9
**Women's Agency**						
Wife beating justified score (mean, SD)[2]	-0.8 (3.6)	-2.1 (0.1)	-0.4 (3.9)	-2.0 (3.5)	-0.5 (3.8)	-2.0 (3.5)
Decision maker on respondent's contraceptive use (joint or alone) (%)[3]	77.6	84.7	83.7	85.5	83.1	85.4
**Ecological variables[6]**						
Under-5 mortality rate (mean, SD)					165.8 (12.3)	124.1 (5.9)
Source of family planning supplies (private) (mean, SD)					0.4 (0.1)	0.2 (0.06)

1. A sum of 11 total possible modern methods that respondent knows about (can range from 0 to 11) with a score of 1 being assigned for every modern method known. Methods: (1) female sterilization, (2) male sterilization, (3) contraceptive pill, (4) IUD, (5) injectables, (6) implants, (7) male condom, (8) female condom, (9) diaphragm/contraceptive foam/contraceptive jelly female condom, (9) lactational amenorrhea method, (10) standard days method, and (11) emergency contraception.
2. Varies from -5 to +5 based on responses to five questions about beating justification; a higher score indicates more agreement that beating is justified and a lower score means less agreement that beating is justified
The individual is given a score of: (-1) if beating is not justified for particular question; (0) if response is 'don't know'; (1) if beating is justified. The five questions considered for beating justification: 1. If wife goes out without telling husband; 2. If wife neglects children; 3. If wife argues with husband; 4. If wife refuses to have sex with husband; 5. If wife burns the food
3. Only asked among partnered women, so the %'s are the same as the partnered women's table.

2.2 Married/In-Union Women

Table 2. Descriptive Statistics of Women included in the Married/In-Union Women Analyses

**Survey year**	**2008**	**2019**	**2008**	**2019**	**2008**	**2019**
**Eligibility Criteria[1]**						
Currently married or in union (%)	49.1	30.4	87.3	82.7	74.9	62.4
Had sex in past 12 months (%)	72.8	81.6	76.5	83.7	75.4	83
Not currently pregnant (%)	90	93.4	92.8	94	91.9	93.8
Not menopausal (%)	99.9	99.9	95.2	93.6	96.8	96.1
Fecund (%)	96.5	99.3	95.6	95.5	95.9	97
Total eligible (N) - ***unweighted***	541	1,039	2,564	5,526	3,105	6,565
**Outcome: Current use of any modern contraceptive method (%)**	5.7	28.5	9.5	30	8.8	29.8
**Sociodemographic characteristics**						
Respondent's age (in years) (mean, SD)	20.6 (2.4)	21.3 (2.1)	33.9 (6.5)	34.9 (6.5)	31.6 (7.8)	32.8 (7.8)
Respondent's years of education (mean, SD)	1.5 (3.0)	5.2 (4.4)	1.8 (3.7)	2.9 (4.5)	1.7 (3.5)	3.2 (4.6)
Wealth Index (1-10) (mean, SD)	4.5 (3.0)	5.1 (3.1)	4.8 (3.1)	5.3 (3.2)	4.8 (3.1)	5.3 (3.2)
Age at first sex (mean, SD)	14.9 (2.1)	15.2 (1.9)	15.7 (2.8)	15.7 (2.2)	15.5 (2.7)	15.6 (2.2)
Recent employment (%)	77.9	75.1	87.1	87.8	85.5	85.8
Urban residence (%)	27	40.1	30.2	39.5	29.6	39.6
**Family Planning and Health Systems Interventions**						
Source of family planning for users						
**Public** – gov't clinic/pharmacy, gov't home/community delivery (%)	59	81.5	59.5	84.7	59.4	84.3
**Private** - Private clinic/delivery, pharmacy (%)	41	18.5	40.5	15.3	40.6	15.7

**Family Planning methods ever heard of**						
**Modern methods (mean, SD)[2]**	2.3 (2.2)	7.3 (2.5)	2.7 (2.4)	7.5 (2.5)	2.6 (2.4)	7.5 (2.5)
Contraceptive Pill (%)	54.8	96.8	61.2	96.8	60.1	96.8
Intrauterine Device (IUD) (%)	19.7	70	25.5	72.8	24.5	72.3
Injectables (%)	51.4	96.7	60.9	96.6	59.2	96.6
Implants (Norplant) (%)	2.5	95.9	3.6	96.3	3.4	96.2
Diaphragm (%)	NA	NA	NA	NA	NA	NA
Foam and Jelly (%)	2.3	NA	3.3	NA	3.1	NA
Male Condom (%)	55.4	95.2	57.3	95.1	57	95.1
Female Condom (%)	12.1	67.3	15.9	66.6	15.2	66.7
Female Sterilization (%)	15.8	61.2	24.1	65	22.6	64.4
Male Sterilization (%)	3.4	30.1	7.2	31.2	6.5	31.1
Emergency Contraception (%)	4.2	37.7	5.3	38	5.1	37.9
Lactational Amenorrhea Method (LAM) (%)	7.3	51.5	8.9	56.4	8.6	55.7
Standard Days Method (SDM)/Cycle Beads (%)	NA	32.2	NA	32.9	NA	32.8
Other Modern Method (%)	0	3.3	0	4.8	0	4.5
**Traditional Methods**						
Periodic Abstinence (Rhythm) (%)	7.3	38.9	10.1	38.7	9.6	38.7
Withdrawal (%)	9.2	59.1	10.9	56.6	10.6	57
Other Traditional Methods (%)	37.2	13.6	39.8	16.1	39.3	15.7
**Current modern methods used**						
Contraceptive Pill (%)	45.3	12.6	39.7	21.5	40.4	20.1
IUD (%)	2.9	1.2	3.5	1.9	3.5	1.8
Injectables (%)	26.1	49.6	47.1	42	44.7	43.1
Implants (%)	0	35.3	0	32.1	0	32.6
Diaphragm (%)	NA	NA	NA	NA	NA	NA
Foam and Jelly (%)	NA	NA	NA	NA	NA	NA
Male condom (%)	16.6	0.5	6.9	0.6	8	0.6
Female condom (%)	0	0	0	0.06	0	0.05
Female sterilization (%)	0	0	0	1	0	0.9
Male sterilization (%)	0	0	0	0.04	0	0.04
Emergency Contraception (%)	NA	0.4	NA	0.4	NA	0.4
(LAM) (%)	9.1	0.4	2.7	0.08	3.5	0.1
SDM/Cycle Beads (%)	NA	0	NA	0.3	NA	0.3
Other modern method (%)	0	0	0	0	0	0
**Reasons for contraceptive method discontinuation (%)**						
Became pregnant while using	NA	1.4	NA	1.9	NA	1.8
Wanted to become pregnant	NA	29.7	NA	30.8	NA	30.6
Husband/partner disapproved	NA	10.1	NA	4.4	NA	5.4
Side effects	NA	42.5	NA	43.9	NA	43.7
Health concerns	NA	0	NA	0	NA	0
Access, availability (lack of access/too far)	NA	2.7	NA	1.2	NA	1.5
Wanted more effective method	NA	3.8	NA	5.5	NA	5.2
Inconvenient to use	NA	4.1	NA	4.1	NA	4.1
Infrequent sex, husband away	NA	1	NA	3.2	NA	2.8
Cost too much	NA	2.2	NA	1.4	NA	1.6
Fatalistic	NA	0	NA	0.4	NA	0.3
Difficult to get pregnant/ menopausal	NA	0	NA	0.3	NA	0.2
Marital dissolution/Separation	NA	0	NA	0.1	NA	0.09
Other	NA	2.1	NA	2.5	NA	2.5
Don't know	NA	0.5	NA	0.2	NA	0.2

**Contacts with healthcare**						
Visited by family planning worker in past 12 months (%)	7.5	31.4	8.3	29.5	8.2	29.8
Visited health facility in past 12 months (%)	33.8	62.8	36.7	55.9	36.2	57
**Recent exposure to family planning messages in last 12 months (%)**						
On radio	39.5	30.9	44.9	31.2	44	31.1
In newspaper	3.3	1.8	4.5	2.2	4.3	2.1
On TV	4.7	9.7	5.7	11	5.5	10.8
**Exposure to mass media**						
Frequency of reading newspaper or magazine (%)						
not at all	96.7	93.8	92	94.2	92.8	94.1
< once a week	1.4	5.1	3.3	3.7	3	3.9
at least once a week	1.7	1.1	3.8	2.2	3.4	2
Almost everyday	0.2	NA	0.9	NA	0.8	NA
Frequency of listening to radio (%)						
not at all	49.5	58.2	43.9	54.4	44.9	55
< once a week	10.7	20	9.9	18.9	1	19.1
at least once a week	17.5	21.9	20.4	26.6	19.9	25.9
Almost everyday	22.3	NA	25.8	NA	25.2	NA
Frequency of watching television (%)						
not at all	87.2	75.2	88.1	74.7	88	74.8
< once a week	5.5	13	3.9	11.9	4.1	12.1
at least once a week	4.2	11.8	5	13.4	4.8	13.1
Almost everyday	3.1	NA	3.1	NA	3.1	NA
**Getting medical help for self (issues deemed a big problem)**						
Getting permission to go (%)	7.8	23.7	7.1	24.9	7.2	24.7
Getting money for treatment (%)	81.6	67	81.1	68.5	81.2	68.3
Distance to health facility (%)	57.1	42.1	56.1	45.8	56.3	45.2
Not wanting to go alone (%)	21.8	19.6	19.7	22	20	21.6
**Women's Agency**						
Wife beating justified score (mean, SD)[3]	-0.2 (3.7)	-1.6 (3.5)	-0.2 (3.9)	-1.8 (3.6)	-1.4 (4.1)	-1.8 (3.5)
Decision making power (mean, SD)[4]	0.1 (1.8)	-0.3 (1.8)	0.3 (1.7)	-0.1 (1.8)	0.3 (1.8)	-0.1 (1.8)
Decision maker on large household purchases (joint or alone) (%)	45.2	43.9	52.6	48.2	51.3	47.6
Decision maker on visits to family or relatives (joint or alone) (%)	57.8	41.9	63.3	47.2	62.4	46.4
Decision maker on what to do with husband's earnings (joint or alone) (%)	NA	NA	NA	NA	NA	NA
Decision maker on food to be cooked each day (joint or alone) (%)	NA	NA	NA	NA	NA	NA
Decision maker on respondent's own healthcare (joint decision or alone) (%)	45.6	41.6	54.5	45	53	44.5
Decision maker on respondent's contraceptive use (joint or alone) (%)	77.6	84.7	83.7	85.5	83.1	85.4
Education difference (in years) between respondent and partner (mean, SD)	-1.1 (4.0)	-1.1 (5.0)	-1.5 (4.1)	-1.3 (4.9)	-1.4 (4.1)	-1.3 (4.9)
Age difference (in years) between respondent and partner (mean, SD)	-12.5 (9.5)	-10.7 (11.4)	-10.9 (9.1)	-9.7 (10.2)	-11.7 (9.2)	-9.9 (10.4)
Age at first cohabitation (in years) (mean, SD)	15.7 (2.6)	16.9 (2.6)	17.4 (4.7)	19.4 (5.4)	17.1 (4.4)	19.0 (5.1)
**Ecological Variables[7]**						
Under-5 mortality rate (mean, SD)[8]					165.8 (12.3)	124.1 (5.9)

Source of family planning supplies (private) (mean, SD)					0.4 (0.1)	0.2 (0.06)

1. Eligibility criteria percentages for partnered women are the same as All Women *except currently married or in union (%) and the Total Eligible N*
2. A sum of 11 total possible modern methods that respondent knows about (can range from 0 to 11) with a score of 1 being assigned for every modern method known. Methods: (1) female sterilization, (2) male sterilization, (3) contraceptive pill, (4) IUD, (5) injectables, (6) implants, (7) male condom, (8) female condom, (9) diaphragm/contraceptive foam/contraceptive jelly female condom, (9) lactational amenorrhea method, (10) standard days method, and (11) emergency contraception
3. Varies from -5 to +5 based on responses to five questions about beating justification; a higher score indicates more agreement that beating is justified and a lower score means less agreement that beating is justified
The individual is given a score of: (-1) if beating is not justified for particular question; (0) if response is 'don't know';
(1) if beating is justified. The five questions considered for beating justification: 1. If wife goes out without telling husband; 2. If wife neglects children; 3. If wife argues with husband; 4. If wife refuses to have sex with husband; 5. If wife burns the food.
4. Each item (listed below) was assigned (-1) if the decision was made by the husband or other alone and (1) if the decision was made jointly or by the respondent alone. A low score means low decision-making power and a high score means decision-making power. Large household purchases, visiting family/relatives (range: -2 to 2).

2.3 Oaxaca-Blinder Decomposition

2.3.1 All Women Ages 15-49-Years-Old, N=13,819

All Women Ages 15-49-Years-Old, N=13,819				
**Factors**	**Estimated coefficient**	**Mean difference (2019-2008)**	**Predicted change in mCPR**	**Share of predicted change in (%)**
**Outcome: Current use of modern contraception**		0.243	0.093	38.30%
Level 3 variables				
Respondent's years of education (continuous)	0.012	2.044	0.024	25.80%
Relationship status (0=other, 1=married/in-union)	-0.081	-0.12	0.01	10.40%
Urban residence (0=rural, 1=urban)	0.059	0.106	0.006	6.80%
Employed in last 12 months (0=no, 1=yes)	0.043	-0.028	-0.001	-1.30%
Getting permission a barrier to seeking medical help for self (0=no, 1=yes)	-0.046	0.159	-0.007	-7.80%
Respondent's decision-making power (ecological from 3 decisions)	0.204	-0.086	-0.017	-18.70%
Level 4 variables				
# of modern FP methods ever heard of (range: 0-10)	0.014	4.359	0.063	67.20%
Visited by fieldworker in past 12 months (0=no, 1=yes)	0.071	0.2	0.014	15.20%
Visited a health facility in past 12 months (0=no, 1=yes)	0.06	0.167	0.01	10.70%
Recent exposure to FP messages on TV (0=no, 1=yes)	-0.055	0.059	-0.003	-3.50%
Recent exposure to FP messages on radio (0=no, 1=yes)	0.029	-0.152	-0.004	-4.80%

There was a 24.3% increase in mCPR amongst women ages 15-49. The decomposition model predicted 9.3% of this change, and the factors entered into the model explained 38.3% of the predicted change. The mean increase in knowledge of modern methods by 4.36 points explained most, 67.2%, of the predicted change in mCPR over time. This was followed by the increase in mean years of education (2.0) explaining 25.5% of the predicted change, and then by the 20% increase in recent fieldworker visits explaining 15.2% of the change. Recent exposure to FP messages on TV was negatively associated with mCPR (beta coefficient: -0.055) while exposure to FP messages on the radio was positively associated (beta coefficient: 0.029), but decreased over time by 15.2%, thus explaining a negative share of the change.

2.3.2 All Women Ages 15-24-Years-Old, N=4,303

All Women Ages 15-24-Years-Old, N=4,303				
**Factors**	**Estimated coefficient**	**Mean difference (2019-2008)**	**Predicted change in mCPR**	**Share of predicted change in (%)**
**Outcome: Current use of modern contraception**		0.298	0.171	57.50%
Level 3 variables				
Respondent's years of education (continuous)	0.019	3.194	0.061	35.40%
Relationship status (0=other, 1=married/in-union)	-0.174	-0.202	0.035	20.50%
Level 4 variables				

# of modern FP methods ever heard of (range: 0-10)	0.014	4.271	0.061	35.70%
Visited a health facility in past 12 months (0=no, 1=yes)	0.095	0.15	0.014	8.30%

There was a 29.8% increase in mCPR amongst women ages 15-24. The decomposition model predicted 17.1% of this change, and the factors entered into the model explained 57.5% of the predicted change. The mean increase in knowledge of modern methods by 4.27 points explained most, 35.7%, of the predicted change in mCPR over time. This was followed by the increase in mean years of education (3.19) explaining 35.4% of the predicted change, and then by relationship status which explained 20.5% of the change. Being married or in-union was negatively associated with mCPR and the prevalence of 15-24-year-olds who were married decreased by 20.2%. Because the share of predicted change in mCPR is the product of the estimated coefficient and the change over time, relationship status explains a positive share of the predicted change.

2.3.3 All Women Ages 25-49-Years-Old, N=9,522

All Women Ages 25-49-Years-Old, N=9,522				
**Factors**	**Estimated coefficient**	**Mean difference (2019-2008)**	**Predicted change in mCPR**	**Share of predicted change in (%)**
**Outcome: Current use of modern contraception**		0.212	0.084	39.60%
Level 3 variables				
Respondent's years of education (continuous)	0.008	1.321	0.011	12.50%
Urban residence (0=rural, 1=urban)	0.056	0.104	0.006	6.90%
Relationship status (0=other, 1=married/in-union)	-0.031	-0.048	0.001	1.80%
Getting permission to seek medical help for self a big problem (0=no, 1=yes)	-0.059	0.167	-0.01	-11.80%
Respondent's decision-making power (ecological from 3 decision variables)	0.258	-0.086	-0.022	-26.40%
Level 4 variables				
# of modern FP methods ever heard of (range: 0-10)	0.016	4.392	0.069	82.70%
Visited by fieldworker in past 12 months (0=no, 1=yes)	0.083	0.206	0.017	20.40%
Visited a health facility in past 12 months (0=no, 1=yes)	0.053	0.178	0.009	11.10%
Recent exposure to FP messages via newspaper (0=no, 1=yes)	-0.073	-0.032	0.002	2.80%

There was a 21.2% increase in mCPR amongst women ages 25-49. The decomposition model predicted 8.4% of this change, and the factors entered into the model explained 39.6% of the predicted change. The mean increase in knowledge of modern methods by 4.39 points explained most, 82.7%, of the predicted change in mCPR over time. This was followed by increased visits by fieldworkers explaining 20.4% of the predicted change, and then by an increase in years of completed education explaining 12.5% of the predicted change. High decision-making power was positively associated with mCPR among older women, however decision-making power decreased over time at the ecological level, resulting in decision-making power explaining a negative share of the predicted change.

2.3.4 Partnered Women Ages 15-49-Years-Old, N=9,298

Partnered Women Ages 15-49-Years-Old, N=9,298				
**Factors**	**Estimated coefficient**	**Mean difference (2019-2008)**	**Predicted change in mCPR**	**Share of predicted change in (%)**
**Outcome: Current use of modern contraception**		0.209	0.097	46.60%
Level 3 variables				
Respondent's years of education (continuous)	0.013	1.531	0.02	20.10%
Urban residence (0=rural, 1=urban)	0.052	0.1	0.005	5.30%
Education difference (continuous; respondent's-partner's)	-0.005	0.112	-0.001	-0.60%
Respondent participates in decision making for own healthcare (0=no, 1=yes)	0.049	-0.084	-0.004	-4.20%
Getting permission to seek medical help for self a big problem (0=no, 1=yes)	-0.048	0.174	-0.008	-8.60%
Age at first cohabitation (continuous)	-0.006	1.947	-0.011	-11.70%
Level 4 variables				
# of modern FP methods ever heard of (range: 0-10)	0.015	4.484	0.066	67.70%
Visited by fieldworker in past 12 months (0=no, 1=yes)	0.091	0.216	0.02	20.10%
Visited a health facility in past 12 months (0=no, 1=yes)	0.046	0.207	0.01	9.80%
Recent exposure to FP messages via newspaper (0=no, 1=yes)	-0.089	-0.022	0.002	2.00%

Among partnered women ages 15-49, mCPR increased by 20.9%. The decomposition model predicted 9.7% of this change, and the factors entered into the model explained 46.6% of the predicted change. The mean increase in knowledge of modern methods by 4.48 points explained most, 67.7%, of the predicted change in mCPR over time. The increase in mean years of education (1.53) and percent of women visited by a fieldworker (21.6%) each explained 20.1% of the predicted change.

2.3.5 Partnered Women Ages 15-24-Years-Old, N=1,524

Partnered Women Ages 15-24-Years-Old, N=1,524				
**Factors**	**Estimated coefficient**	**Mean difference (2019-2008)**	**Predicted change in mCPR**	**Share of predicted change in (%)**
**Outcome: Current use of modern contraception**		0.228	0.151	66.50%
Level 3 variables				
Respondent's years of education (continuous)	0.02	3.632	0.073	48.00%
Urban residence (0=rural, 1=urban)	0.103	0.132	0.014	8.90%
Any child loss (0=no, 1=yes)	-0.07	-0.013	0.001	0.60%
Respondent participates in decision making (three decisions; -3 to 3 higher score = respondent participates)	0.012	-0.439	-0.005	-3.40%
Level 4 variables				
# of modern FP methods ever heard of (range: 0-10)	0.011	4.733	0.053	34.90%
Visited by fieldworker in past 12 months (0=no, 1=yes)	0.069	0.24	0.017	10.90%

Among partnered women ages 15-24, mCPR increased by 22.8%. The decomposition model predicted 15.1% of this change, and the factors entered into the model explained 66.5% of the predicted change. The mean increase in years of education (3.63) explained the largest share, 48.0%, of predicted change, followed by a 4.73 point increase in knowledge of modern methods explaining 34.9% and recent visits by a fieldworker explaining 10.9% of the predicted change.

2.3.6 Partnered Women Ages 25-49-Years-Old, N=7,793

Partnered Women Ages 25-49-Years-Old, N=7,793				
**Factors**	**Estimated coefficient**	**Mean difference (2019-2008)**	**Predicted change in mCPR**	**Share of predicted change in (%)**
**Outcome: Current use of modern contraception**		0.205	0.083	40.60%
Level 3 variables				
Respondent's years of education (continuous)	0.011	1.133	0.012	14.90%
Urban residence (0=rural, 1=urban)	0.04	0.093	0.004	4.50%
Education difference (continuous; respondent's-partner's)	-0.005	0.161	-0.001	-1.00%
Respondent participates in decision making for own healthcare (0=no, 1=yes)	0.052	-0.095	-0.005	-5.90%
Getting permission to seek medical help for self a big problem (0=no, 1=yes)	-0.056	0.177	-0.01	-12.10%
Age at first cohabitation (continuous)	-0.006	2.064	-0.012	-13.90%
Level 4 variables				
# of modern FP methods ever heard of (possible range: 0-10)	0.014	4.428	0.062	74.90%
Visited by fieldworker in past 12 months (0=no, 1=yes)	0.094	0.211	0.02	23.90%
Visited a health facility in past 12 months (0=no, 1=yes)	0.05	0.19	0.009	11.40%
Distance to facility is a big problem to seek medical help for self (0=no, 1=yes)	-0.025	-0.103	0.003	3.10%

Among partnered women ages 25-49, mCPR increased by 20.5%. The decomposition model predicted 8.3% of this change, and the factors entered into the model explained 40.6% of the predicted change. The mean increase in knowledge of modern methods by 4.43 points explained most, 74.9%, of the predicted change in mCPR over time. The increase in percent of women visited by a fieldworker (21.6%) explained 23.9% of the predicted change, and the increase in mean years of education (1.13) explained 14.9% of the predicted change.

2.3.7 Summary of Top 3 Drivers by Analysis

All ages (15-49)	All women	1st	# of modern FP methods ever heard of
		2nd	Respondent's years of education
		3rd	Decision-making power
	Partnered	1st	# of modern FP methods ever heard of
		2nd	Respondent's years of education
		3rd	Visited by fieldworker in past 12 months
Younger women (15-24)	All women	1st	# of modern FP methods ever heard of
		2nd	Respondent's years of education
		3rd	Visited health facility in past 12 months
	Partnered	1st	Respondent's years of education
		2nd	# of modern FP methods ever heard of
		3rd	Visited by fieldworker in past 12 months
Older women (25-49)	All women	1st	# of modern FP methods ever heard of
		2nd	Decision-making power
		3rd	Visited by fieldworker in past 12 months
	Partnered	1st	# of modern FP methods ever heard of
		2nd	Visited by fieldworker in past 12 months
		3rd	Respondent's years of education
