# Supplementary material for: Progress in family planning in Sierra Leone: a mixed-methods case study
Source: BMJ Glob Health. 2026 Jun 9;11(Suppl 3):e018775. doi: 10.1136/bmjgh-2024-018775 (PMC13250227; doi:10.1136/bmjgh-2024-018775)
Supplement: online supplemental file 6 [file bmjgh-11-Suppl_3-s007.docx]

**Supplementary File 6. Mapping pathways from family planning policy and programme bundles (1990–2023) to study conceptual framework domains and modern contraceptive prevalence rate (mCPR) gains in Sierra Leone**

| **Name of policy/programme bundle (Era)** | **National context** | **Government Commitment** | **FP Financing, policy, and programmes** | **Sub-national/local context** | **Individual factors** | **Women’s agency** |
| --- | --- | --- | --- | --- | --- | --- |
| **Foundation & Wartime Resilience (1990–2001)** United Nations Population Fund (UNFPA) Country Programmes (1971–); first Marie Stopes clinic and media campaign (1988); government adoption of population and development agendas (e.g. Dakar/Ngor Declaration (1992), 1994 International Conference on Population and Development); National Population Policy (1988/1989). | UN technical support, capacity-building, and high-level advocacy strengthened institutional leadership and established a supportive, rights-based climate for family planning; built political and social will; positioned population and gender in national development discourse through alignment with international treaties. | Formal policies declaring reproductive health and family planning (RH/FP) a national priority galvanised political and donor support, setting mandates and enabling cross-sector action; instituted first national population and health policies prioritising family planning; established foundational commissions and units to guide future expansion. | Policies established the supply chain as a priority and enabled NGO/private sector provision (Marie Stopes, PPASL), securing commodity flow and building a wide service delivery network for better access and choice of method; introduced donor funding and technical support (mainly UNFPA); launched National Family Planning Programme and first integrated NGO services; early adoption of mass communication strategies. | Introduced community-level and peri-urban/urban outreach; trained community leaders and traditional birth attendants; adapted messaging and services to local needs, even during conflict; mass media (radio, billboards, posters) promoted family planning**.** | Programmes built knowledge, attitudes and perceptions towards HIV and family planning (FP); task-sharing and community involvement increased provider capacity, confidence and relevance; targeted FP education improved access and awareness for women and couples; promoted modern contraceptive use among early adopters, including displaced and refugee populations. | Enabled some women to seek and use FP services; increased knowledge and self-efficacy; small but significant steps towards autonomy and shifting norms. |
| **Post-War Reconstruction & Gender Empowerment (2002–2007)**  National HIV/AIDS Policy (2002, Act 2007); Education Act (2004); Local Government Act (2004); United Nations Cooperation Framework (2004–07); World Bank SHARP (2002–07); Child Rights Act (2007); launch of National Gender Mainstreaming Policy and programmes; expansion of UNFPA programming. | Prioritised peace, rebuilding and gender equality; international commitments (e.g. MDGs, UNSCR 1325) and TRC recommendations created momentum to address the rights and needs of women and youth. | New policies mainstreamed gender equity, children’s rights and HIV prevention in law and ministry action; expanded mandate for reproductive and sexual health education and services in schools and communities. | Major donor and UN funding (DFID, FCDO, UNCF, World Bank SHARP, Global Fund, UNFPA) enabled programme expansion, condom programming, supply of RH commodities, provider training, and advocacy; HIV/AIDS prevention and family planning messages were integrated. | Decentralised services; community-based AIDS committees; school-based reproductive health education and teacher training; local by-law enforcement and livelihood support for women and ex-combatants; rehabilitation of health infrastructure. | Expanded school and community outreach, sexuality education, and service access for women and girls; BCC, IEC, and peer outreach for HIV prevention and family planning; strengthened knowledge, shifted norms, and increased rights awareness. | New laws and school policies protected rights, promoted leadership, and reduced early marriage and violence; increased girls’ schooling, training, and legal protection expanded women’s decision-making power and autonomy |
| **Fee Removal & Systems Strengthening (2008–2013)**  Agenda for Change (2008–2012); Free Health Care Initiative (FHCI, 2010); UN Joint Programme for Reproductive and Child Health (2008–2010); UNFPA Country Programmes for family planning and Global Programme for Reproductive Health Commodity Security (2008– ); Restless Development Youth Reproductive Health Programme (2007–2012), funded by Foreign, Commonwealth & Development Office (FCDO); Health System Supply Chain Reform (NPPU Act, 2012); FCDO-funded Integrated Reproductive, Maternal and Newborn Health (IRMNH) programme (2012–2016); National Education Policy (2010); Community Health Worker Policy (2012); National Strategy for Reduction of Teenage Pregnancy (2013– ); World Bank Performance-Based Financing (2013–2017). | Political transition and high-level commitment to reducing maternal and child mortality; presidential leadership on reproductive health; promotion of girls’ education and gender equality; donor alignment; focus on MDGs, SDGs and poverty reduction**.** | Free health care (FHCI) for women and under-five children; integration of family planning into sector plans; new adolescent and youth health and education policies; gender acts; community health worker policies; increased focus on the basic package of essential health services. | Major increases in government and pooled donor funding for maternal health, including family planning; performance-based financing targeting mCPR; expanded supply chain; scaled-up outreach and continuous funding for commodity security; increased donor and NGO support for teenage pregnancy reduction, women’s empowerment, and gender equity initiatives. | Rapid PHU expansion and CHW scale-up; extended outreach to rural and marginalised communities; integration with schools and community organisations; expanded mass media campaigns; increased NGO and government-led family planning service delivery in rural areas. | Increased youth and girls’ access to services and information; higher contraceptive knowledge and use; delayed marriage; empowerment through livelihoods and education; increased community involvement. | Strengthened legal protections and women’s decision-making; increased community and facility support for contraceptive uptake; more contraceptive options; more women informed and empowered to access family planning services leading to increased uptake. |
| **Equity, Adolescents, & Strategic Innovations (2015–2023):**  Full integration of family planning into Basic Package of Essential Health Services (2015–2020); FCDO Saving Lives in Sierra Leone Project (2016–2023) funding FHCI; Six-Monthly Contact Point (6MlyCP, 2017); RMNCAH/RCH Strategies (2017–2021); Family Planning Costed Implementation Plan (2018–2023); National Population Policy (2018); FP2020 and FP2030 commitments; comprehensive sexuality education (CSE) in schools (2019); UNFPA Transformative Results Plan (2020–2023); Radical Inclusion in Schools Policy (2021). | Post-Ebola recovery prioritised health system resilience, digital data/monitoring and evaluation, and equity; alignment with international and donor priorities, including SDGs and FP2020 commitments; intensified adolescent and last-mile focus; increased commitment to girls’ education**.** | Rights-based legal frameworks; formalised costed family planning policy; integration of comprehensive sexuality education, gender equality, adolescent-friendly services, and inclusive education (e.g. Girls’ Access to Education) into sectoral policies. | Large-scale donor (UNFPA, FCDO, UNICEF, WHO) and government financing and technical support; expanded supply chain reforms (NMSA); digital last-mile tracking; adolescent-focused service packages; NGO and partner (Marie Stopes, PPASL) programme scale-up. | Community health worker and peer educator scale-up; school- and community-based outreach; targeted programmes for rural and underserved groups; increased service points and mobile initiatives. | Greater family planning knowledge, reduced stigma, increased adolescent and youth uptake; social marketing, life skills and targeted comprehensive sexuality education expanded demand and contraceptive use among marginalised groups. | Enhanced agency through legal rights, skills-building, safe spaces, male engagement and digital peer support; empowered women and girls to access and use family planning services and assert reproductive choices; increased contraceptive prevalence and demand satisfied. |

**Abbreviations:** BCC, behaviour change communication; CSE, comprehensive sexuality education; CHW, community health worker; DFID, Department for International Development (UK); FCDO, Foreign, Commonwealth & Development Office (UK); FHCI, Free Health Care Initiative; FP, family planning; GPRHCS, Global Programme for Reproductive Health Commodity Security (UNFPA); IEC, information, education and communication; ICPD, International Conference on Population and Development; IRMNH, Integrated Reproductive, Maternal and Newborn Health programme; MDGs, Millennium Development Goals; mCPR, modern contraceptive prevalence rate; NGO, non-governmental organisation; NMSA, National Medical Supply Agency (Sierra Leone); NPPU Act, National Pharmaceutical Procurement Unit Act (2012); PHU, peripheral health unit; PPASL, Planned Parenthood Association of Sierra Leone; RH/FP, reproductive health/family planning; RMNCAH/RCH, reproductive, maternal, newborn, child and adolescent health / reproductive and child health strategies; SDGs, Sustainable Development Goals; SHARP, Sierra Leone HIV/AIDS Response Project (World Bank); TBAs, traditional birth attendants; TRC, Truth and Reconciliation Commission; UNCF, United Nations Cooperation Framework; UNFPA, United Nations Population Fund; UNICEF, United Nations Children’s Fund; UNSCR 1325, United Nations Security Council Resolution 1325 on Women, Peace and Security; WHO, World Health Organization.
